# Methylome Diversification through Changes in DNA Methyltransferase Sequence Specificity

**DOI:** 10.1371/journal.pgen.1004272

**Published:** 2014-04-10

**Authors:** Yoshikazu Furuta, Hiroe Namba-Fukuyo, Tomoko F. Shibata, Tomoaki Nishiyama, Shuji Shigenobu, Yutaka Suzuki, Sumio Sugano, Mitsuyasu Hasebe, Ichizo Kobayashi

**Affiliations:** 1 Department of Medical Genome Sciences, Graduate School of Frontier Sciences, University of Tokyo, Minato-ku, Tokyo, Japan; 2 Institute of Medical Science, University of Tokyo, Minato-ku, Tokyo, Japan; 3 National Institute for Basic Biology, Okazaki, Japan; 4 Advanced Science Research Center, Kanazawa University, Kanazawa, Japan; 5 Department of Basic Biology, School of Life Science, Graduate University for Advanced Studies, Okazaki, Japan; Indian Institute of Science, India

## Abstract

Epigenetic modifications such as DNA methylation have large effects on gene expression and genome maintenance. *Helicobacter pylori*, a human gastric pathogen, has a large number of DNA methyltransferase genes, with different strains having unique repertoires. Previous genome comparisons suggested that these methyltransferases often change DNA sequence specificity through domain movement—the movement between and within genes of coding sequences of target recognition domains. Using single-molecule real-time sequencing technology, which detects N6-methyladenines and N4-methylcytosines with single-base resolution, we studied methylated DNA sites throughout the *H. pylori* genome for several closely related strains. Overall, the methylome was highly variable among closely related strains. Hypermethylated regions were found, for example, in *rpoB* gene for RNA polymerase. We identified DNA sequence motifs for methylation and then assigned each of them to a specific homology group of the target recognition domains in the specificity-determining genes for Type I and other restriction-modification systems. These results supported proposed mechanisms for sequence-specificity changes in DNA methyltransferases. Knocking out one of the Type I specificity genes led to transcriptome changes, which suggested its role in gene expression. These results are consistent with the concept of evolution driven by DNA methylation, in which changes in the methylome lead to changes in the transcriptome and potentially to changes in phenotype, providing targets for natural or artificial selection.

## Author Summary

Living organisms are affected by epigenetic variation in addition to DNA sequence variation. DNA methylation is one of the most studied epigenetic modifications in both prokaryotes and eukaryotes. In prokaryotes, most DNA methylation is by DNA methyltransferases with high sequence specificity. *Helicobacter pylori*, a human stomach pathogen responsible for stomach cancer and other diseases, carries a large number of DNA methyltransferase genes that vary among strains. In this work, we examined the distribution of DNA methylation in multiple *H. pylori* genomes using single-molecule real-time sequencing technology, which detects DNA methylation with single-base resolution. Comparison of methylation motifs between closely related genomes allowed assignment of a recognition sequence to each DNA methylation specificity-determining gene. Highly methylated genes were detected, although the general DNA methylation pattern varied among strains. Knockout of a methylation specificity-determining gene led to changes in the transcriptome. These findings are consistent with our hypothesis that changes in the methylome lead to changes in the transcriptome and to changes in phenotypes, providing potential targets for natural and artificial selection in adaptive evolution.

## Introduction

Epigenetic modifications affect gene regulation and genome maintenance [1], [2]. DNA methylation is an important epigenetic modification in bacteria with functions in gene expression regulation, genome replication initiation, cell cycle regulation, anti-mutagenesis, and genome maintenance [3], [4]. Eukaryotes use a few DNA methyltransferases to mainly methylate DNA at CpG and other low-specificity sequences. In bacteria, DNA is methylated by a variety of DNA methyltransferases, most of which have high sequence specificity. Methylations in both promoter and coding regions affect gene expression [2], [5], [6]. Methyltransferases are often members of restriction-modification (RM) systems [7] and are called modification (M) enzymes.

**Figure 1:**
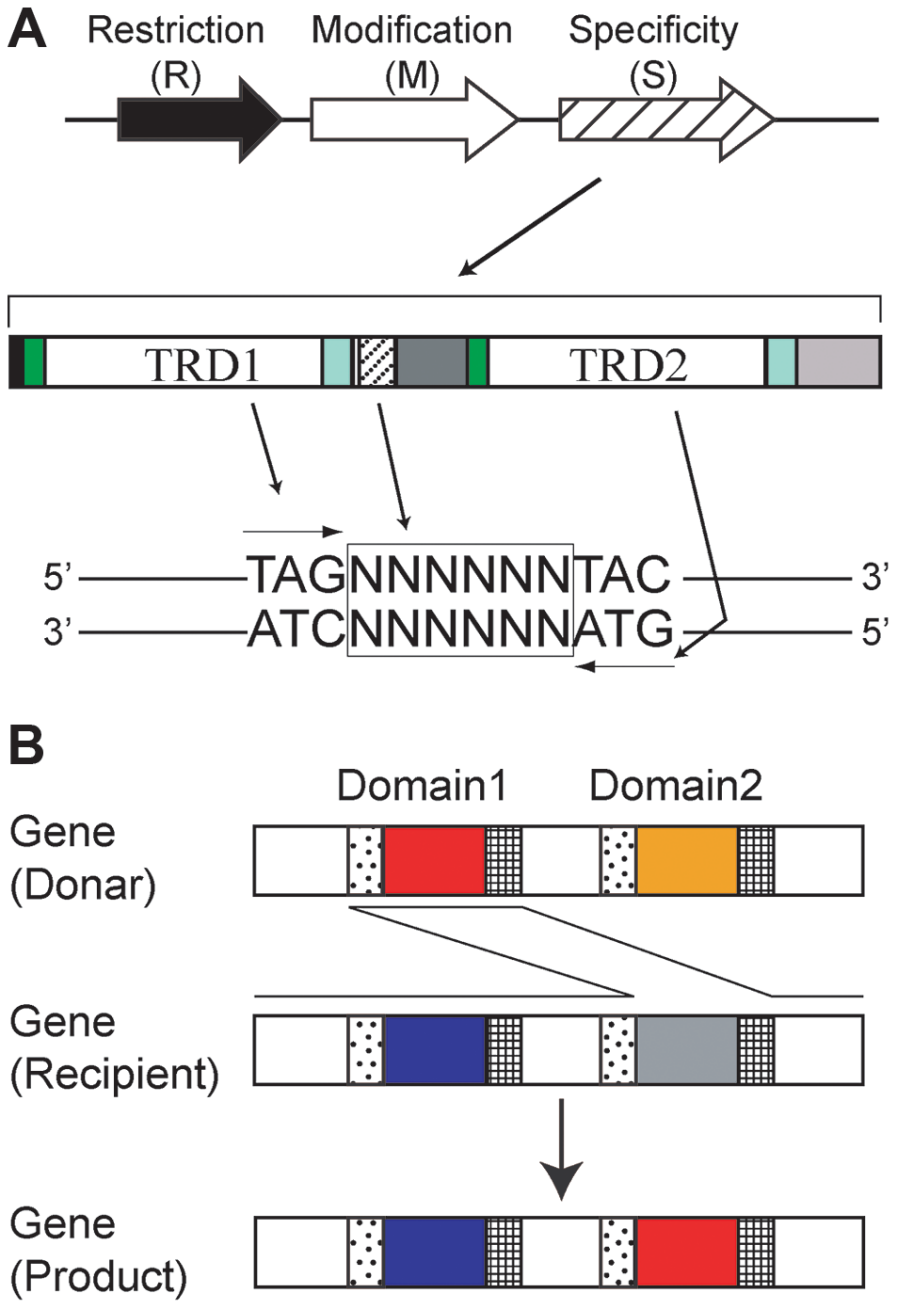
Type I RM system. (A) A Type I RM system consists of restriction (R), modification (M) and specificity (S) genes. A specificity gene typically encodes two target recognition domains (TRD) in tandem. Each domain recognizes half of a bipartite target sequence. Copy number of repeat sequences between the two domains in some S genes determines the number of Ns (N = A, T, G or C) in the middle of their DNA target sequence. A TRD that recognizes a particular target sequence will recognize the reverse complement sequence when moved to the other TRD site. A TRD sequence that recognizes 5′-TAG-3′/3′-ATC-5′ duplex at the TRD1 site recognizes 5′-CTA-3′/3′-GAT-5′ when moved to the TRD2 site. (B) Domain Movement (DoMo). Amino acid sequences move between TRD1 and TRD2 in the same gene (locus) likely through recombination at repeat sequences flanking TRD1 and TRD2.

Three types of methyltransferases, corresponding to RM systems Type I through III, are known [8]. A Type II methyltransferase such as M.EcoRI methylates a base within a recognition sequence that is often palindromic [7]. Several classes of Type II methyltransferases recognize nonpalindromic sequences [9]. In living cells, DNA methylation protects the genome from cleavage by a cognate restriction enzyme such as R.EcoRI, which recognizes the same sequence as its cognate methyltransferase [10]. Type III methyltransferases recognize nonpalindromic sequences and methylate only one of the two strands of the recognition sequence [11]. In a Type I RM system, an M gene product forms a complex with its specificity (S) gene product to define the recognition sequence for methyltransferase activity (Figure 1) [12].

RM systems are often mobile and vary among bacterial species and strains [13], [14]. *Helicobacter pylori*, a gastric bacterium that is pathogenic in humans, has one of the highest numbers of identified M genes [15]. Its genome is highly diverse among strains [16], [17]. Each strain has a unique set of M genes, which suggests variable genomic methylation states or methylomes [15].

In addition to the divergent repertoire of M genes, *H. pylori* genome comparisons suggest that Type I and Type III target recognition domain (TRD) sequences are themselves mobile, leading to variation in the DNA sequences recognized by these RM systems. [18]–[20]. TRDs of Type III systems even move between nonorthologous genes by recombination at weakly similar DNA sequences encoding conserved amino acid motifs of DNA methyltransferases; this mechanism spreads TRDs beyond species boundaries [19]. The Type I S protein carries two TRDs, TRD1 and TRD2 (Figure 1), with each domain recognizing half of a bipartite recognition sequence [12]. Not all but some of the Type I S genes show tandem repeat sequences flanked by the two TRDs [18], and their copy number correlates with the length of the central nonspecific region (Ns) in the recognition sequence [21]. TRD sequences of Type I S genes can be shuffled at each domain site (TRD1 and TRD2), leading to diversity in methylation sequence specificity [22]–[27]. Similar amino acid sequences that recognize similar DNA sequences were found in TRD1 of one Type I RM system and in TRD2 of another Type I RM system from a different bacterial species [27]. For *H. pylori* and several other bacteria, a genome comparison revealed that TRD sequences likely move between TRD1 and TRD2 by recombination at their flanking repeat sequences, in a process called Domain Movement (DoMo) [18]. TRD amino acid sequences in Type I S genes and Type III M genes in *H. pylori* fall into distinct homology groups. Amino acid sequences are nearly identical within each group and are expected to correspond to a unique set of recognition sequences. Therefore, the movements of TRD sequences will lead to changes in their recognition sequences. TRD sequence movements along with allelic recombination events, point mutations, and changes in the copy number of tandem repeats between TRD1 and TRD2 are expected to be sources of methylome diversity in *H. pylori* [14]. We hypothesized that such methylome changes might lead to changes in the transcriptome and cell phenotypes and contribute to adaptive evolution [14], [20].

Recognition sequences are known only for some *H. pylori* RM systems, mostly for Type II methyltransferases [28], [29]. For Type II and Type III systems, recognition sequences can be determined by cleaving a DNA molecule of a known sequence and identifying the sequence common to every cut site. However, this method cannot be applied to Type I systems because the corresponding restriction enzyme complex cleaves DNA at unpredictable positions outside the recognition sequence [12]. Their recognition sequences have been determined by transfer of labeled methyl groups by the methyltransferase [30], [31] and transformation by plasmids with or without their candidates [32].

The recent development of single-molecule real-time (SMRT) sequencing technology has facilitated detection of methylated DNA bases [33]. This technology uses a single DNA polymerase to incorporate one of four fluorescent analogs for dATP, dTTP, dGTP, and dCTP onto a DNA template, and monitors the incorporation to decode the template sequence. This technology allows detection of several base modifications in the template DNA because the modifications delay incorporation of the dNTP analog. This method has been established as reliable for accurately detecting methylation motifs in plasmids and bacterial genomes [34]–[39], but no study has been reported that uses this method on more than three closely related bacterial strains.

In this study, we decoded the methylome of closely related *H. pylori* strains and compared their methylomes. We verified a correspondence between genes, TRD sequences and recognition sequences for Type I, II and III systems and found that DoMo in Type I S genes indeed changed recognition sequences and the methylome. Furthermore, transcriptome analysis revealed that methylation by a Type I S protein affected gene expression.

## Results

### Methylome decoding in closely related strains

We analyzed five *H. pylori* strains (P12, F16, F30, F32 and F57) and two isogenic P12 derivatives (HPYF1 and HPYF2, see below). The complete genome sequences of the first five strains were obtained by the Sanger method [17], [40]. P12 was isolated in Germany [40] and belongs to hpEurope cluster in the current population assignment based on STRUCTURE analysis of 7 housekeeping genes [17]. F16, F30, F32 and F57, isolated from the same hospital in Japan [17], [40], fall into the hspEAsia of the hpEastAsia cluster [17], [41]. Their genome sequences are closely related [17], [41], although their synteny has changed through multiple inversion events [42].

A PacBio RS (Pacific Biosciences) was used for SMRT sequencing for methylome analysis. Two biological replicates were analyzed for each strain (DRA accession no. DRA001084). Results were reproducible for output read numbers and for detection of methylated motifs (Table S1). Around 45,000 to 80,000 bases per genome were detected as methylated (N6-methyladenine and N4-methylcytosine, Figure S1). The genome of all strains is around 1.6 Mbp [17], [40], so 1.4 to 2.6% of bases were methylated (with consideration of both strands). Thus, these genomes represent the most heavily methylated genomes analyzed so far by SMRT technology [35]–[37].

**Figure 2:**
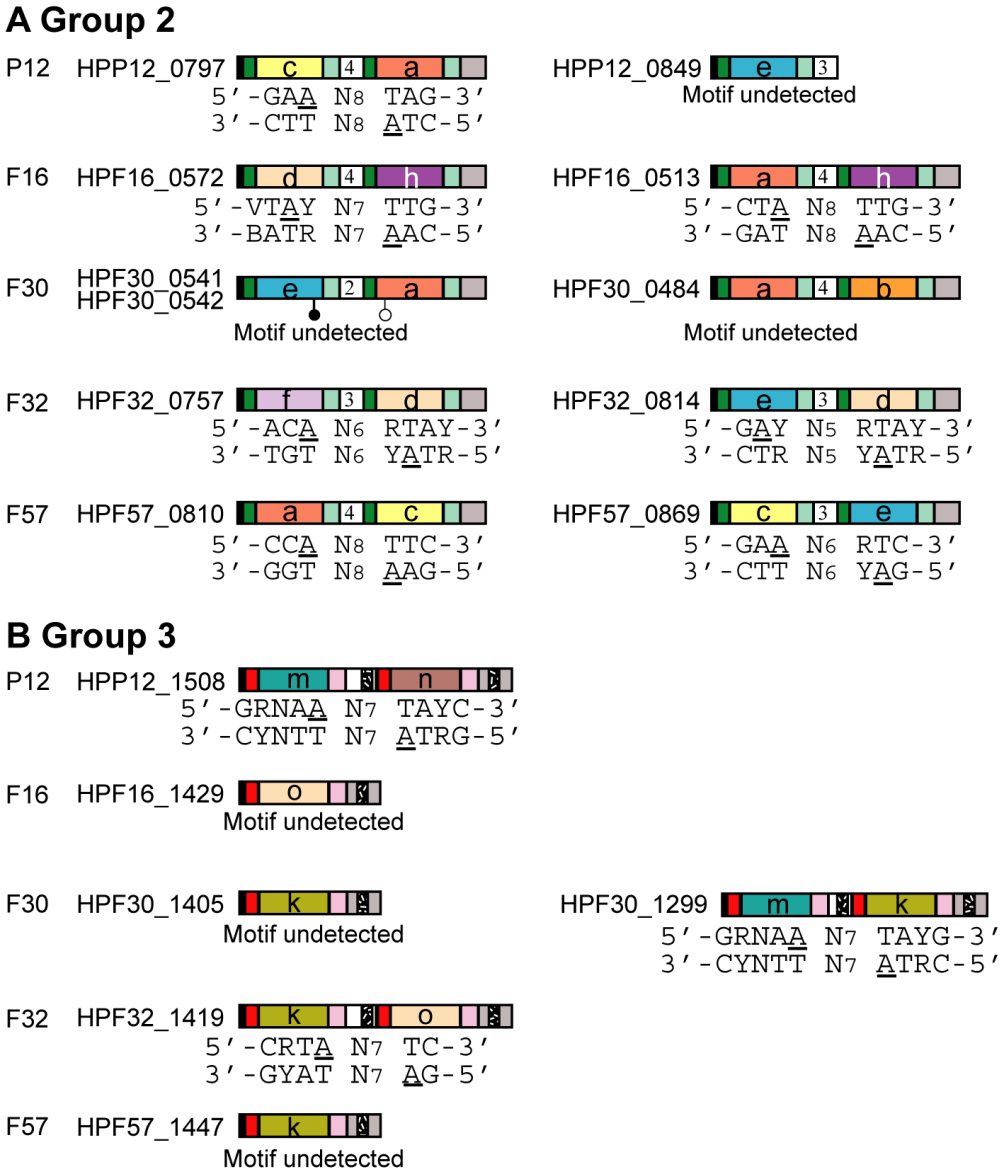
Assignment of a methylation motif to target recognition domains of Type I specificity genes. (A) Group 2 Type I S genes. (B) Group 3 Type I S genes. Amino acid sequences in target recognition domains were classified into homology groups represented by different colors (a, b, c, d, e, f, h, k, m, n, o) [18]. Symbols are as in Figure 1. The number in the middle of the gene is the copy number of tandem repeats. Underline, adenine nucleotides within motifs detected as methylated; white circle, start codon in an unusual position; black circle, stop codon in an unusual position.

Motif search between 20 bp upstream and downstream of each methylated base identified 15 to 24 unique methylation motifs (with variation among strains), with methylation of more than 20% of the copies of each methylation motif in a genome in both biological replicates (Figure 2, Table 1, Table 2, Table S2). In addition to simple 4 bp, 5 bp and 6 bp palindromic methylation motifs, we found nonpalindromic methylation motifs. We also found bipartite methylation motifs that include a long tract of Ns (N = A, T, G or C), which are typical of recognition sequences of Type I RM systems and some subclasses of Type II systems.

**Table 1:** Fraction of methylated copies along a genome for each known Type II methyltransferase (M) genes.

		Strains									
		P12		F16		F30		F32		F57	
Motif^a^	Gene name	Locus tag^b^	%^c^	Locus tag	%	Locus tag	%	Locus tag	%	Locus tag	%
5'-**ATTAAT**	M.hpyAVII	HPP12_0488	**93**	HPF16_0459	**98**	HPF30_0843	**86**	HPF32_0853	**97**	HPF57_0507	**95**
5'-**TCNNGA**	-	HPP12_1052	**95**	HPF16_1028	**99**	HPF30_0302	**96**	HPF32_1023	**98**	HPF57_1048	**98**
5'-**CATG**	M.hpyAI	HPP12_1173	**85**	HPF16_1145	**94**	HPF30_0185	**87**	HPF32_1140	**92**	HPF57_1170	**90**
5'-**GATC**	M.hpyAIII	HPP12_0095	**97**	HPF16_0105	**100**	HPF30_1171	**97**	HPF32_0104	**99**	(HPF57_0142)	**5** d
										(HPF57_0143)	
5'-**GTAC**	M.hpyAXII	HPP12_0510	**97**	HPF16_0851	**99**	-	**7** e	HPF32_0484	**99**	HPF57_0534	**98**
5'-**GANTC**	M.hpyAIV	(HPP12_1318)	**6** d ^,^ f	HPF16_0047	**100**	-	**6** d ^,^ g	HPF32_0049	**99**	HPF57_0049	**99**
5'-**GAATTC**	-	HPP12_1389	**95**	-	**0**	HPF30_1285	**91**	-	**0**	-	**3**
5'-**GAAGG**	M.hpyAV	HPP12_0048	**96**	-	**9** h	(HPF30_1247)	**12** i	-	**0**	-	**5** j
5'-**GATGG**	-	-	**5** d ^,^ f	HPF16_1396	**100**	HPF30_1367	**94**	HPF32_1386	**99**	-	**6** d
5'-**CTNAG**	M.hpyHI	-	**0**	HPF16_0320	**96**	HPF30_0982	**90**	-	**0**	HPF57_0366	**94**
5'-**CCNNGG**	M.hpy99IV	-	**0**	HPF16_0699	**87**	-	**2**	-	**5** k	-	**5** k
5'-**GRRGA**	M1.hpyAII	-	**19** f ^,^ l ^,^ m	-	**8** l ^,^ n ^,^ o	-	**11** l ^,^ m ^,^ o	HPF32_0034	**91**	-	**8** m ^,^ n
5'-**TCTTC**	M2.hpyAII	-	**0**	-	**0**	-	**0**	HPF32_0033	**88**	-	**0**
5'-**CTRYAG**	-	-	**0**	-	**16** p	-	**16** p	-	**13** p	HPF57_1130	**99**
5'-**GAGG**	M1.hpyAVI	HPP12_0044	**95**	*HPF16_0057*	***7*** d q	HPF30_1250	**96**	HPF32_0059	**98**	HPF57_0060	**97**
5'-**GTNNAC**	M.hpyAIX	*HPP12_0908*	***10*** p	HPF16_0891	**100**	HPF30_0429	**97**	HPF32_0444	**99**	*HPF57_0920*	***28*** p
5'-**TCGA**	M.hpyAX	*HPP12_0259*	***37*** l	HPF16_0267	**99**	HPF30_1036	**97**	HPF32_0269	**98**	(HPF57_0278)	**20** n
										(HPF57_0312)	
5'-**TGCA**	-	*HPP12_1523*	***10*** r	HPF16_1447	**99**	HPF30_1424	**95**	HPF32_1440	**98**	HPF57_1467	**97**
5'-**CCGG**	-	*HPP12_0262*	***1***	HPF16_0270	**91**	*HPF30_1033*	***43***	HPF32_0272	**87**	HPF57_0316	**89**

a Methylated base is underlined.

b Locus tag of a truncated gene, which has shorter than 80% of the intact ORF, is bracketed. Locus tag with rare methylated sites is in italics.

c Average in two biological replicates.

d Methylated copies overlap with 5′-TCNNGA and 5′-GAYN_6_TTC.

e Methylated copies overlap with 5′-GTNNAC.

f Methylated copies overlap with 5′-GNGRGA.

g Methylated copies overlap with 5′-TCGA .

h Methylated copies overlap with 5′-CGRAG.

i Methylated copies overlap with 5′-CNNGNAG.

j Methylated copies overlap with 5′-GAAN_6_RTC.

k Methylated copies overlap with 5′-CCGG.

l Methylated copies overlap with 5′-GATC.

m Methylated copies overlap with 5′-GAGG.

n Methylated copies overlap with 5′-GANTC.

o Methylated copies overlap with 5′-GATGG.

p Methylated copies overlap with 5′-TGCA and 5′-GTAC.

q Methylated copies overlap with 5′-GCRGA.

r Methylated copies overlap with 5′-CATG.

**Table 2:** Assignment of a target sequence to each TRD in Type I specificity (S) genes.

Group^a^	TRD homology group^b^	TRD Length (aa)	Locus tag^e^	Recognition sequence^c^^,^ d
Group 2	a	140	HPP12_0797, HPF16_0513	5′-CTA
			HPF57_0810	5′-CCA
	b	146	HPF30_0484	N/D^f^
	c	123	HPP12_0797, HPF57_0810	5′-GAA
	d	126	HPF32_0814, HPF32_0757	5′-RTAY
			HPF16_0572	5′-VTAY
	e	119	HPF32_0814, HPF57_0869	5′-GAY
	f	122	HPF32_0757	5′-ACA
	h	120	HPF16_0513, HPF16_0572	5′-CAA
Group 3	k	125	HPF30_1299, HPF32_1419	5′-CRTA
	m	142	HPP12_1508, HPF30_1299	5′-GRNAA
	n	121	HPP12_1508	5′-GRTA
	o	113	HPF32_1419	5′-GA

a Type I S genes were classified according to orthologous groups [18].

b Classification based on amino acid sequence similarity[18].

c Adenine detected as methylated is underlined.

d Y, C or T; R, A or G; V, A or C or G; B, C or G or T.

e Locus tag of genes that include the TRD.

f N/D, methylation motif not detected.

Many of the methylation motifs were successfully assigned to M/S genes using previous knowledge about recognition sequences [10], presence or absence of apparently intact and untruncated M/S gene orthologs in genomes, and combinations of TRD amino acid sequences in Type I specificity genes [18].

### Methylation hot and cold spots

**Figure 3:**
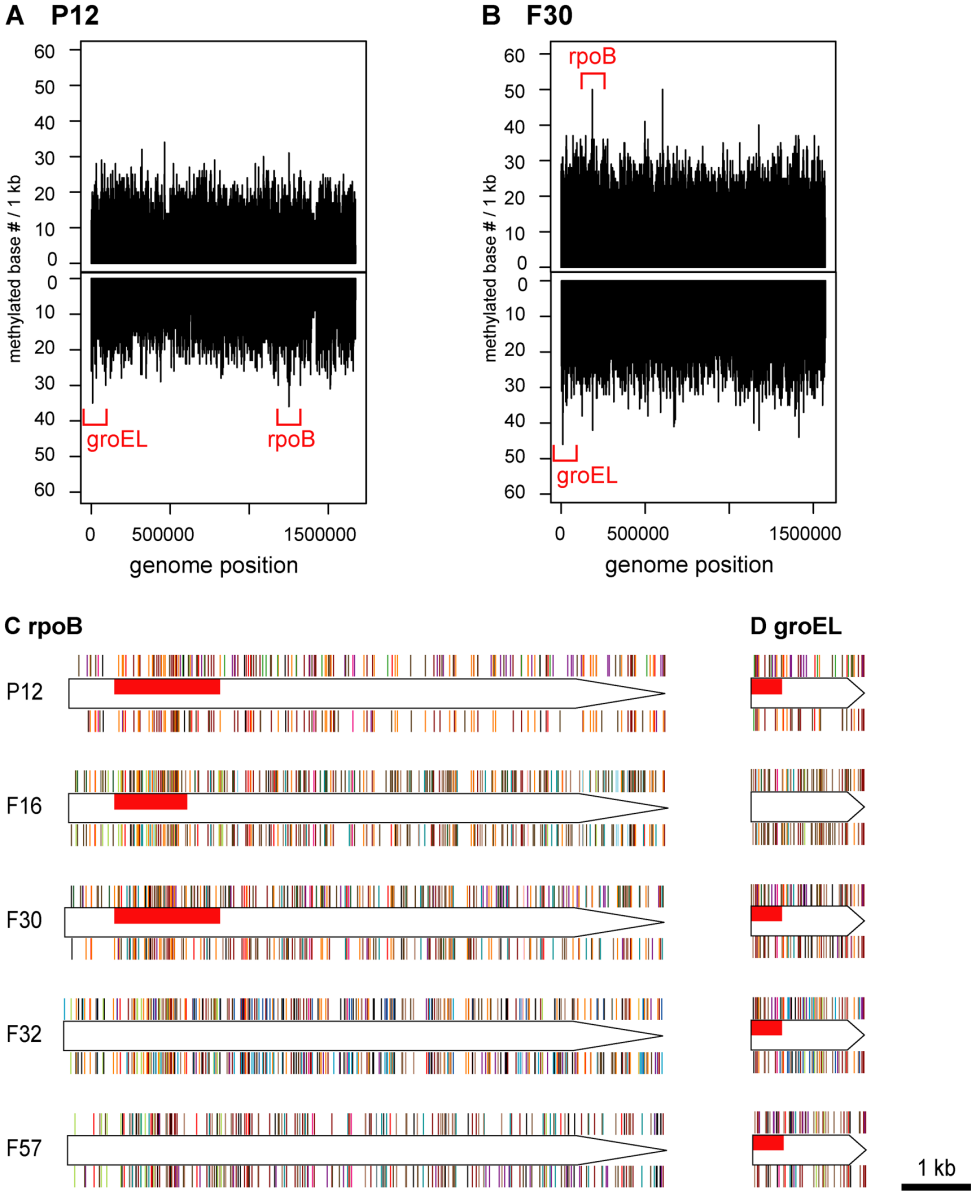
Distribution of methylated bases in genomes and hypermethylated genes. (A) Density of methylated bases on each strand of the P12 genome. (B) Density of methylated bases on each strand of the F30 genome. See Figure S2 for the other strains. (C) Distribution of methylated bases in *rpoB* of each strain. (D) Distribution of methylated bases in *groEL* of each strain. Red box, regions with the densest methylation within the gene. Bar color indicates a specific methylation motif. See Figure S3 for color and distribution of each methylation motif.

To identify hypermethylated or hypomethylated genomic regions, the number of detected methylated bases per 1 kb was calculated for each strand. The five most and least methylated regions for each strain were determined (Table 3, Table 4, Figure 3, Figure S2). A region within the *rpoB* gene, encoding the RNA polymerase beta subunit, was identified as a densely methylated region in three of the five strains (P12, F16, F30). A region within the *groEL* gene, encoding a chaperonin, was also identified as densely methylated and was in the top 5 densely methylated regions in four of the five strains (P12, F30, F32, F57). Both regions were especially heavily methylated at 5′-CATG (methylated base is underlined, Figure S3). The M gene with this recognition sequence (M.hpyAI) is highly conserved among the five strains (Table 1, Table S2) [43] and likely responsible for the conservation of the hypermethylated state.

**Table 3:** Top 5 hypermethylated regions in each strain.

Strain	Methylated base #	Start	End	Strand	Locus tag	Orientation	Annotation
P12	36	1251001	1252000	-	HPP12_1163	sense	DNA-directed RNA polymerase subunit beta rpoB
	35	8501	9500	-	HPP12_0008	sense	chaperonin groEL
	34	463501	464500	+	HPP12_0447	sense	DNA methylase
	32	320501	321500	+	HPP12_0305	sense	glutamate-1-semialdehyde aminotransferase hemL
	32	1251501	1252500	-	HPP12_1163	sense	DNA-directed RNA polymerase subunit beta rpoB
F16	55	1209501	1210500	-	HPF16_1134	sense	DNA-directed RNA polymerase subunit beta rpoB
	52	501001	502000	+	HPF16_0494	sense	flagellar hook protein flgE
	52	1043001	1044000	-	HPF16_0987	sense	biotin sulfoxide reductase bisC fragment
	50	1042501	1043500	-	HPF16_0986	sense	biotin sulfoxide reductase bisC fragment
	49	86001	87000	-	HPF16_0084	sense	urease subunit alpha ureC
F30	50	603501	604500	+	HPF30_0567	sense	hypothetical protein
	50	185501	186500	+	HPF30_0196	sense	DNA-directed RNA polymerase subunit beta rpoB
	48	186001	187000	+	HPF30_0196	sense	DNA-directed RNA polymerase subunit beta rpoB
	46	8501	9500	-	HPF30_0008	sense	chaperonin groEL
	44	1411501	1412500	-	HPF30_1330	sense	hypothetical protein
F32	51	685001	686000	-	HPF32_0628	sense	N-methylhydantoinase
	49	1204501	1205500	-	HPF32_1126	sense	elongation factor G fusA
	47	546501	547500	-	HPF32_0506	sense	cag pathogenicity island protein cagY
	46	8501	9500	-	HPF32_0008	sense	chaperonin groEL
	46	33501	34500	+	HPF32_0030	sense	rod shape-determining protein mreB
F57	44	1351001	1352000	-	HPF57_1280	sense	hypothetical protein
	42	85001	86000	-	HPF57_0083	sense	urease subunit alpha ureC
	41	1176501	1177500	-	HPF57_r03	sense	16S ribosomal RNA
	41	8501	9500	-	HPF57_0008	sense	chaperonin groEL
	39	1351501	1352500	-	HPF57_1281	sense	fumarate hydratase fumC

**Table 4:** Top 5 hypomethylated regions in each strain.

Strain	Methylated base #	Start	End	Strand	Locus tag	Orientation	Annotation	Genomic Islands
P12	1	1419501	1420500	+	HPP12_1351	antisense	integrase/recombinase xercD family protein	TnPZ
	1	1397001	1398000	-	HPP12_1325	antisense	hypothetical protein	TnPZ
	1	478001	479000	+	HPP12_0456	antisense	hypothetical protein	TnPZ
	2	1474501	1475500	+	HPP12_1394	antisense	type IV secretion system ATPase virB11-4	
	2	1421501	1422500	+	HPP12_1353	antisense	relaxase virD2-2	TnPZ
F16	7	400001	401000	-	HPF16_0396	antisense	hypothetical protein	
	9	1373001	1374000	-	HPF16_1309	antisense	hypothetical protein	
	9	1080501	1081500	+	HPF16_1024	antisense	outer membrane protein homC	
	9	1027001	1028000	-	HPF16_0974	antisense	hypothetical protein	
	9	908001	909000	+	HPF16_0870	sense	virulence factor mviN	
F30	2	546501	547500	-	HPF30_0507	sense	hypothetical protein	
	3	546501	547500	+	HPF30_0507	antisense	hypothetical protein	
	5	846001	847000	+	-	-	Intergenic region	
	6	301501	302500	-	HPF30_0293	sense	putative outer membrane protein	
	6	587001	588000	+	HPF30_0549	antisense	hypothetical protein	
F32	3	1082001	1083000	-	HPF32_1015	antisense	hypothetical protein	TnPZ
	5	1076501	1077500	-	HPF32_1009	antisense	hypothetical protein	TnPZ
	6	518001	519000	+	HPF32_0484	sense	Type II modification enzyme	
	7	1085501	1086500	+	HPF32_1019	antisense	outer membrane protein homC	
	7	1082501	1083500	-	HPF32_1015	antisense	hypothetical protein	TnPZ
F57	4	1384501	1385500	-	HPF57_1314	antisense	hypothetical protein	
	5	305001	306000	+	HPF57_0301	antisense	hypothetical protein	TnPZ
	5	305501	306500	+	HPF57_0302	antisense	parA	TnPZ
	5	574501	575500	+	HPF57_0558	antisense	cag pathogenicity island protein cagX	cagPAI
	5	680001	681000	-	HPF57_0648	sense	UDP-N-acetylmuramate—L-alanine ligase murC	

Other genes that were hotspots were central to translation (*fusA*, 16S ribosomal RNA), cell shape determination (*mreB*), or related to host interaction/virulence (*flgE, ureC, cagY*) (Table 3). Relationships were not clear between the hypermethylation in *cagY* gene and its unusual DNA structure with frequent rearrangements leading to gain/loss of function in the Cag type IV secretion system [44]. Hypermethylation in a DNA methyltransferase gene (HPP12_0447) suggested some interaction, such as one in gene expression regulation, between multiple DNA methyltransferases. The *bisC* gene, hypermethylated in an *H. pylori* strain isolated in Japan, appears to be truncated in all hspEAsia strains [17]. The biological signficance of these cases of hypermethylation is not yet clear.

In three strains, P12, F32 and F57, several regions with the lowest methylation (Table 4) were in conjugative transposons, or TnPZs [40], [45]. Fewer methylated sites imply fewer methylation motifs and, therefore, fewer targets for the cognate restriction enzymes. The paucity of methylated motifs and bases might have resulted from selection by restriction attack during horizontal transfer of these elements [46]–[48]. This observation is in contrast to the results from eukaryote genomes, where mobile elements tend to be silenced by methylation [49].

The presence of a hypomethylated region in a Type II modification enzyme gene (HPF32_0484, M.hpyAXII homolog) suggests another type of interaction between multiple DNA methylation systems. We do not know whether its own hypomethylation is related to the mobility found in this family [50] and serves as a means to avoid restriction. Its target sequence (5′-GTAC) is abundant in 16S rRNA gene [51] and contributes to its hypermethylation.

Other hypomethylated genes encoded outer membrane proteins (*homC*, HPF30_0293), a virulence factor (*mviN*), and a cell wall synthesis enzyme (*murC*) (Table 4).

### Methylation activity of Type II M genes with a known recognition sequence

For each of the five studied strains, we compared the list of present or absent M/S genes as annotated in the REBASE database [10] with the list of detected methylation motifs for assignment. The two lists matched well with only few exceptions.

For most of the Type II M genes with a known recognition sequence for N6-methyladenine or N4-methylcytosine, the studied genomes had a high fraction of the copies of the recognition sequence methylated (Table 1). For many of the recognition sequences, around 90% of the copies were methylated. Orthologs of HPP12_0488 (5′-ATTAAT), HPP12_1052 (5′-TCNNGA), and HPP12_1173 (5′-CATG) (Table 1) were highly conserved in all five strains, suggesting a conserved function in this species. As mentioned above, HPP12_1173 orthologs responible for the conserved methylation hotspot in *rpoB* methylation hotspot are highly conserved in *H. pylori* [43]. No Type II-like methylation motifs were newly assigned to M genes with a hitherto unknown motif.

#### Methylation due to an overlapping motif

Even when an ortholog of a predicted M gene was not identified in a genome, a recognition sequence was often methylated at more than 5% of its copies, higher than our false-discovery threshold for methylation detection. All the cases of such methylation without a corresponding M gene could be explained by methylation of an overlapping methylation motif. For example, 5% of 5′-GATC were detected as methylated in strain F57, which lacks an open reading frame (ORF) for the corresponding M gene ortholog (Table 1). The 5′-**GATC** methylation could be explained by methylation activity on 5′-*TC*NN*GA* and 5′-*GAY*NNNNNN*TTC* (Y = C or T), resulting in 5′-*TC*NN***GA*** **TC** and 5′-***GAT*** **C**NNNNN*TTC*. In support of this explanation, all but 14 copies of 5′-**GATC** sites were hemimethylated. The 14 sites that were fully methylated could be explained by recognition sequence overlap on both strands. This result suggested that a low or even an intermediate level of methylation at a motif might be maintained for another overlapping methylation motif. Other cases of overlap are in Table 1.

#### Absence of methylation activity for some M genes

In some cases, methylation activity of an apparently intact, untruncated ORF of an M gene was not detected or detected only rarely. For example, >95% of 5′-GAGG sequences were methylated in 4 strains but only 7% were methylated in strain F16 (Table 1). More than half (173 of 301) of 5′-**GAGG** methylation events in F16 were explained by overlap with 5′-*TC*NN*GA* and 5′-*GCRGA*, resulting in 5′-*TC*NN***GA*** **GG** and 5′-*GCR* ***GA*** **GG**; thus no significant methylation activity could be attributed to the untruncated M gene ORF in F16 (M1.hpyAVI ortholog, HPF16_0057; Table 1).

Other similar cases can also be explained by recognition sequence overlap (Table 1). In F57, 5′-**GT**NN**AC** methylation was explained by 5′-*TGCA* and 5′-*GTAC* methylation, resulting in 5′-**G** ***T*** *GC* ***A*** **C** and 5′-**GT** *GT* ***AC*** . P12 had rare 5′-*TGCA* methylation so that the rare 5′-**GT**NN**AC** methylation in its genome is explained only by 5′-*GTAC* methylation. The 5′-**TCGA** methylation in this strain (37%, Table 1) was explained by 5′-*GATC* methylation, resulting in 5′-**TC** ***GA*** *TC* . The rare 5′-**TGCA** methylation there (10%, Table 1) was explained by 5′-*CATG* methylation, resulting in 5′-**TG** ***CA*** *TG* . Therefore, we concluded that the methylation activity of these ORFs (M.hpyAIX orthologs HPP12_0908 in P12 and HPF57_0920 in F57; M.hpyAX orthologs HPP12_0259 and HPP12_1523 in P12; Table 1) was undetectable.

In several cases, a loss or decrease in methylation activity could be explained by comparing the amino acid sequence encoded by a gene with its active orthologs'. For HPF16_0057, which was expected to methylate 5′-GAGG but appeared to lack this activity, a deletion of 23 amino acids in the N-terminus was observed in addition to strain-specific amino acid changes in conserved amino acid motifs specific to DNA methyltransferases such as P92S and R98C (in the amino acid numbering of HPF16_0057) in motif VIII (Figure S4A). The expression and stability of several DNA methyltransferases are regulated by the N-terminus region [52]. We do not know whether HPF16_0057 was expressed or not. A few strain-specific amino acid changes were detected in other genes in P12 that had apparently intact ORFs but no methylation function, such as V171I in motif VIII of HPP12_0908 (5′-GTNNAC) and A77T in HPP12_0259 (5′-TCGA ) (Figure S4C-D). These deletions and amino acid changes could explain some or all losses or decreases in activity. P12, a European strain, had many nucleotide variations when compared to the four Japanese strains, so it is difficult to identify critical sequence changes without further analysis.

#### Microevolution in methylation motifs

For 5′-CCGG, the fraction of methylated copies varied with the strains (Table 1, the last line). This methylation motif was rarely (1%) methylated in the European strain P12, >87% methylated in three of the four Japanese strains F16, F32, and F57, but 43% methylated in the Japanese strain F30. This intermediate methylation could not be explained by overlap with known methylation motifs. When the methylation of sequences with an additional nucleotide at the 3′-side of 5′-CCGG was analyzed, 5′-CCGGG showed no methylation while 5′-CCGGH (H = A, T or C) was methylated in F30 (Table 5). In brief, equal methylation of 5′-CCGGG and 5′-CCGGH was seen in F16, F32 and F57, both were lost in P12, and stricter sequence specificity was found in F30. P12-specific amino acid changes such as A132T and R181C, and F30-specific amino acid changes, such as P133S and E149A, were found in TRDs of the M ortholog [53] and could explain the differences in activity (Figure S4B).

**Table 5:** Microevolution in methylation motifs.

	Fraction of methylated copies in the genome (%)				
Sequence^a^	P12	F16	F30	F32	F57
5′-CCGG**N**	1	91	43	87	89
5′-CCGG**G**	0	92	0	86	87
5′-CCGG**C**	2	91	54	88	90
5′-CCGG**A**	0	89	36	86	87
5′-CCGG**T**	1	91	62	87	89

a N = A, T, C, G.

### Deduction of recognition sequences of Type I restriction-modification systems

*H. pylori* has three groups of Type I RM systems with a total of at most five Type I S genes at different genetic loci. We defined these as Group 1 through 3 [18]. Type I S genes encode two TRDs, TRD1 and TRD2, in tandem, each binding to half of a bipartite recognition sequence (Figure 1A). For example, in Figure 1A, TRD1 corresponds to the left half 5′-TAG-3′/3′-ATC-5′ whereas TRD2 corresponds to the right half 5′-TAC-3′/3′-ATG-5′. A TRD recognizes half of a target sequence in an inverted configuration when it is at different TRD sites [27].

TRDs in *H. pylori* are classified into homology groups and members of the same homology group have identical or nearly identical amino acid sequences [18]. In the five strains analyzed, TRD homology groups TRD **a**, TRD **b**, TRD **c**, TRD **d**, TRD **e**, TRD **f**, and TRD **h** were identified for Group 2 (Figure 2A); TRD homology groups TRD **k**, TRD **m**, TRD **n**, and TRD **o** were identified for Group 3 (Figure 2B). The combination of TRD homology groups varies among strains [18]. For example, strain P12 carries the combination TRD **c**-TRD **a** (Figure 2A), while strain F16 carries the combination TRD **d**-TRD **h** and the combination TRD **a**-TRD **h** (Figure 2A).

#### Assignment of half-target sequences to TRD homology groups by stepwise comparison

By comparing detected methylation motifs and TRD homology group combinations among the strains, we assigned the half-recognition sequences of Type I systems to the TRD homology groups. For example, the half-motif 5′-**CTA**-3′/3′-**GAT**-5′ was observed in Type I-like methylation motifs in both the P12 and F16 strains: 5′-**CTA**N_8_TTG-3′/3′-**GAT**N_8_ AAC in F16 and 5′-GAAN_8_ **TAG**-3′/3′-CTTN_8_ **ATC**-5′ in P12. Note that, in the two strains, the half-motif is present at different TRD sites (TRD1 *vs.* TRD2) and is regarded as inverted as described above. We assigned the half motif to TRD **a**, the only TRD homology group shared by these two strains.

Next, we used these initial assignments for further assignments. For example, as assigned above, the methylation motif 5′-GAAN_8_TAG-3′/3′-CTTN_8_ ATC-5′ in P12 correspondeds to a S gene with combination of TRD **c** and TRD **a**. Because TRD **a** recognizes the right half of the motif, TRD **c** was assigned to the left half of the motif, 5′-GAA-3′/3′-CTT-5′ (Figure 2A, P12). This assignment was confirmed by detection of TRD **c** and its putative half-motif in two Type I-like methylation motifs in strain F57 (Figure 2A, F57). Using this comparison-based stepwise procedure, we assigned a recognition sequence to each TRD homology group for the S genes of Group 2 and Group 3 (Table 2, Figure 2).

#### Evidence of domain movement

For Group 2 Type I S genes, methylation motifs were assigned to seven ORFs (Figure 2A), resulting in the assignment of half-recognition sequences for six TRD homology groups (Table 2).

For TRD homology groups **a, c, d** and **e**, methylation activity was detected for both of two domain sites (TRD1 and TRD2), strongly suggesting that DoMo (Figure 1B) led to predictable changes in methylation specificity. For example, TRD **a**, which recognized 5′-CTA-3′/3′-GAT-5′, was present in TRD1 in an S gene in strain F16 and in TRD2 in an S gene in strain P12. This result indicated that the TRD homology group members retain methylation activity and have the same sequence specificity at both domain sites.

#### Changes in recognition sequences within TRD homology groups

**Figure 4:**
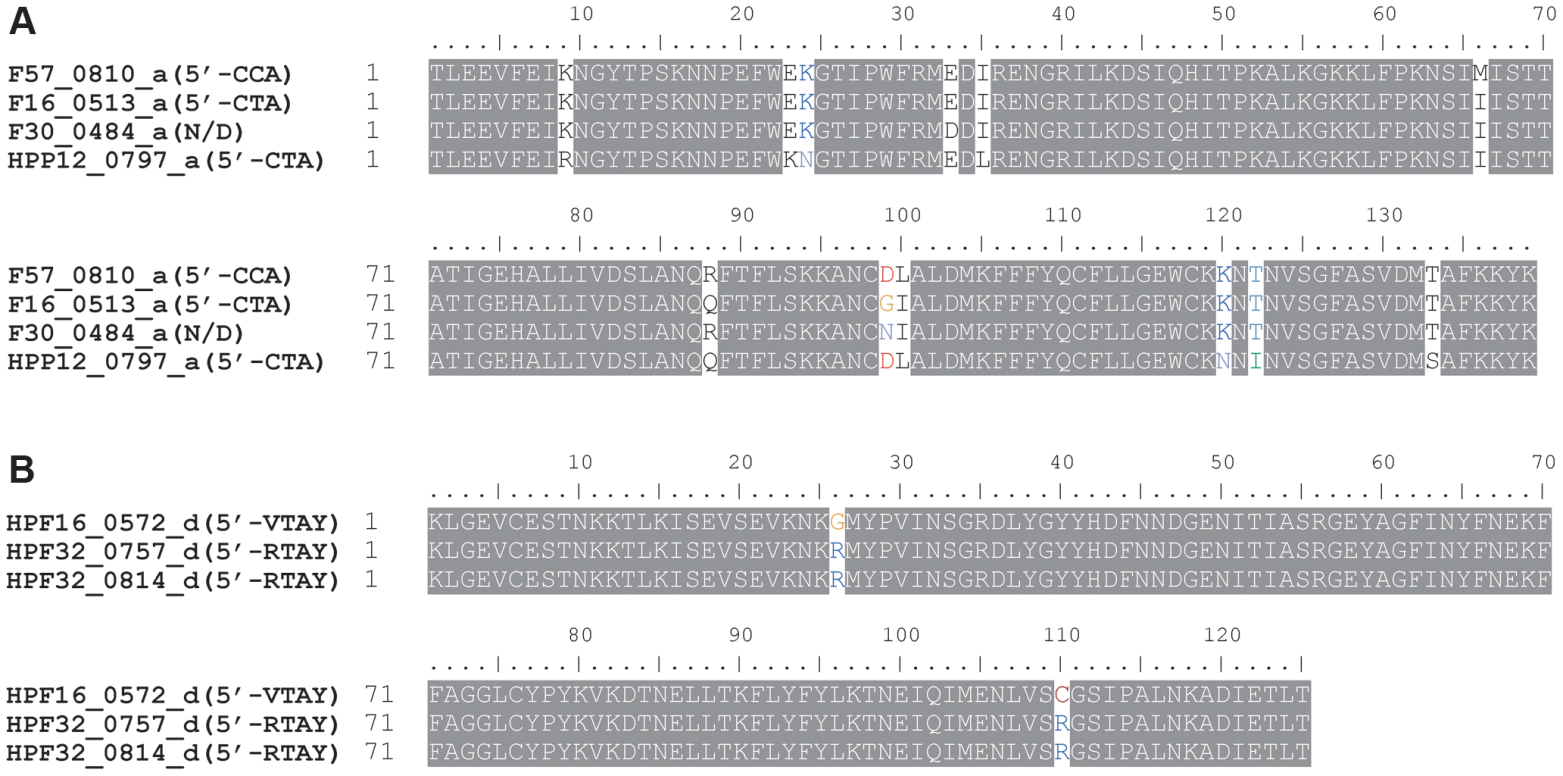
TRD sequences within a homology group differing in the recognition sequence. (A) Homology group **a**. (B) Homology group **d**. Locus tag is followed by TRD homology group name (half recognition sequence). The SNPs co-segregating with the recognition sequence are indicated by fonts in color. N/D, not detected.

In most of the cases, one TRD homology group, classified based on their sequence similarity, corresponds to one recognition sequence. In two TRD homology groups, however, recognition sequences were not identical between strains (Table 2, Figure 2A). The recognition sequence of TRD **a** in HPF57_0810 was 5′-C**C** A-3′/3′-G**G**T-5′, which differed from the 5′-C**T** A-3′/3′-G**A**T-5′ recognized by the TRD **a** members in two other gene products (HPF16_0513, HPP12_0797). The recognition sequence of TRD **d** in HPF16_0572 changed to 5′-**V**TAY-3′/3′-**B**ATR-5′ (V = A, C or G; B = T, G or C) from the palindromic 5′-**R**TAY-3′/3′-**Y**ATR-5′ (R = A or G; Y = T or C) in two methylation motifs in F32 (HPF32_0757, HPF32_0814). Even when the members within each of these TRD groups had different recognition sequences, only few amino acid differences were detected between them (Figure 4A, B). Therefore, we were unable to identify an amino acid identity threshold that could cluster the TRD sequences into homology groups that strictly correspond to recognition sequence differences. This microevolution of sequence recognition through few amino acid changes could be the subject of future studies on DNA sequence recognition by Type I enzymes.

#### Correlation between the TRD length and half-recognition sequence

**Figure 5:**
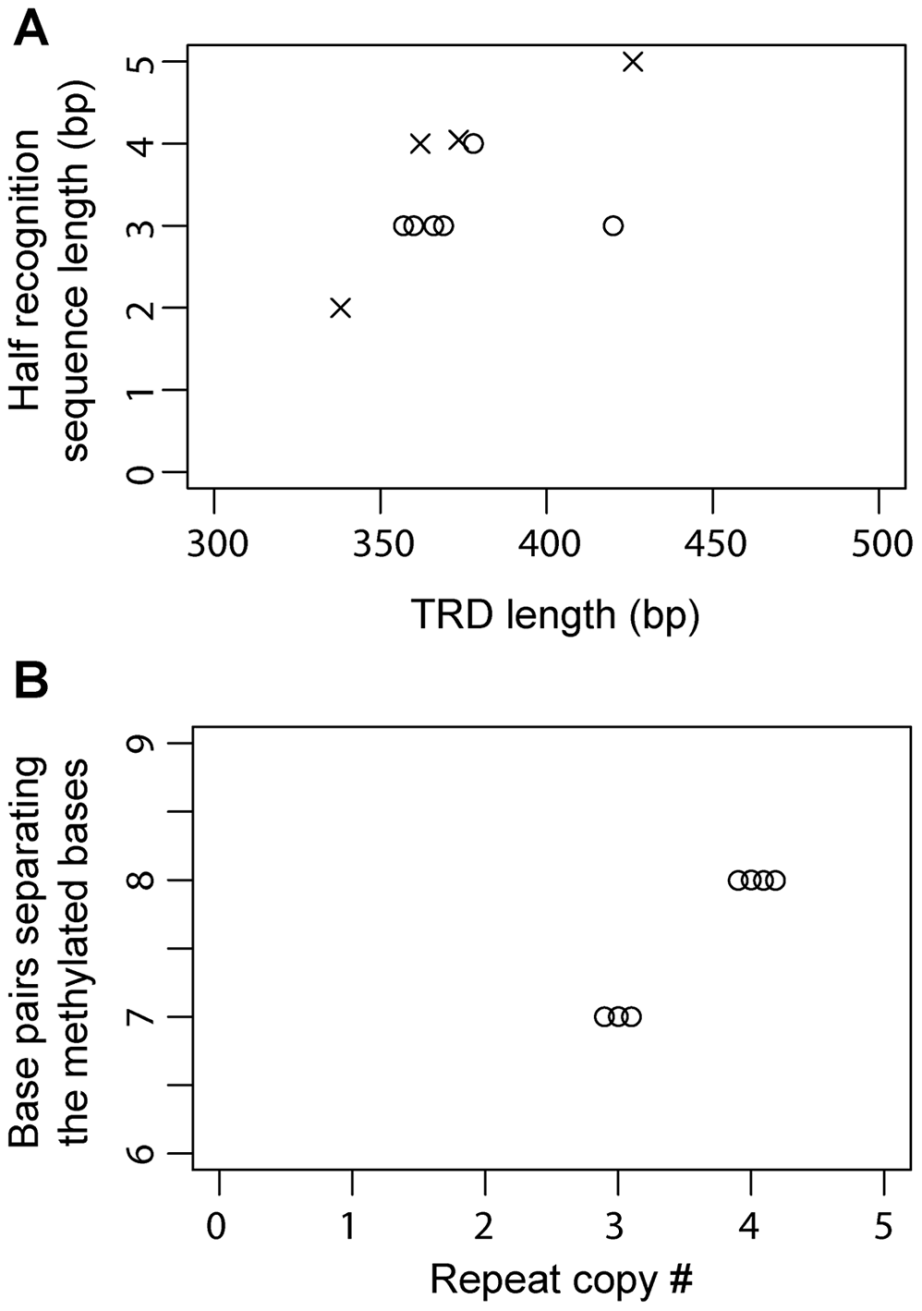
Relationship between TRD structure and recognition sequence in Type I S genes. (A) Plot of the length of TRD1/2 versus the length of half recognition sequence. Circle, Group 2; cross, Group 3. (B) Plot of the copy number of tandem repeats between TRD1 and TRD2 versus the number of base pairs separating the two methylated bases in the full recognition sequences in Group 2.

Half-recognition sequences for the TRDs of Group 2 Type I S genes varied from 3 to 4 bp. Of the six S genes in Group 3, three were assigned methylation motifs, resulting in assignment of four TRD homology groups (Figure 2B). Half-recognition sequences varied from 2 to 5 bp. The 5 bp half-recognition sequence was interrupted by an N ( = A, T, G, or C). TRD **o**, which had a 2 bp recognition sequence, was the shortest of the TRDs, while TRD **m**, with a 5 bp recognition sequence, was the longest. These results suggested a positive correlation between TRD sequence length and half-recognition sequence length (Table 2, Figure 5A).

No methylation motifs were assigned for HPP12_0849, HPF16_1429, HPF30_1405 and HPF57_1447, which had a single TRD site (Figure 2B). Methylation motifs were not detected for HPF30_0484, even though it had an apparently intact and untruncated ORF. A single, strain-specific amino acid change (E33D) was observed in TRD **a** of HPF30_0484 compared to active TRD **a** sequences (Figure 4A), but we do not know whether this change caused enzymatic inactivation.

#### Determinants of base-pair numbers separating bases to be methylated

The number of repeated Ns in the middle of recognition sequences was positively correlated with the number of tandem repeats of amino acid sequences in the middle of related S proteins in some Type I restriction-modification systems (Figure 1A) [21]. We confirmed this relationship within Group 2 S genes. Three central repeats in the coding region resulted in five or six Ns in the recognition sequences and four central repeats resulted in seven or eight Ns. When we plot the repeat copy number versus the number of base pairs separating two methylation bases within a methylation motif, a clear relationship was revealed; 3 central repeats to 7 bp separation and 4 central repeats to 8 bp separation. (Figure 5B). In Group 3, the length of central Ns in the recognition sequences did not vary (N_7_), consistent with the absence of repeats and length changes in the central region of S ORFs.

#### Unassigned Type I-like methylation motifs

Most detected methylation motifs were assigned to a specific M or S gene, but 3 to 9 methylation motifs per genome remain unassigned (Table 6, Table S3). These included Type I-like methylation motifs and nonpalindromic hemimethylated methylation motifs.

**Table 6:** Unassigned methylation motifs in each strain.

Strain	Methylation motif^a^	Fraction of methylated copies		Motif #	Comments
		Sample 1	Sample 2		
A. P12	5′-GCGCGC	21	23	506	
	5′-GNGRGA	94	97	4,015	
	5′-GACC	94	97	2,604	
B. F16	5′-HGATGCAB	56	63	196	
	5′-GATGG	99	100	2,236	
	5′-CGRAG	99	99	1,512	
	5′-GCRGA	99	99	2,327	
	5′-CAGC	99	100	7,175	
C. F30	5′-CANNNNNGTC	95	95	879	
	5′-GACNNNNNTG	94	95	879	Complement with 5′-CANNNNNGTC
	5′-CAAGWAG	46	51	508	Part of 5′-CNNGNAG?
	5′-CRTGHAG	75	77	502	Part of 5′-CNNGNAG?
	5′-CTNGNAG	95	97	1,239	Part of 5′-CNNGNAG?
	5′-CCDGNAG	92	95	733	Part of 5′-CNNGNAG?
	5′-GATGCA	66	60	453	
	5′-AGGAG	95	98	1,456	
D. F32	5′-RGANNNNNNNTCY	99	99	1,854	Palindromic Type I-like methylation motif
	5′-RGANNNNNNNTAY	44	44	2,112	
	5′-RTANNNNNNNTCY	28	28	2,112	Complement with 5′-RGANNNNNNNTAY
	5′-CCTMCA	45	54	837	
	5′-CCRAG	98	99	3,019	
E. F57	5′-CCANNNNNNTAA	96	94	1,229	
	5′-TTANNNNNNTGG	96	93	1,229	Complement with 5′-CCANNNNNNTAA
	5′-RCTANNNNNNTAA	37	34	668	
	5′-TTANNNNNNTAGY	36	32	668	Complement with 5′-RCTANNNNNNTAA
	5′-CCTCTAG	86	95	97	
	5′-GAASC	98	98	4,188	

a Methylated base is underlined.

Assignments could not be made by comparing the remaining Type I-like methylation motifs and the combination of TRD sequences of Type I S genes. For Group 1 Type I S genes [18], one TRD homology group was shared by P12 and F16, and another was shared by F30 and F32. No shared half-motif sequences were identified for each of these TRD pairs. No half-motif sequences were assigned for TRD of Type IIG S genes, either [18]. Such inability to assign might have been due to loss of methylation activity in some combinations of the domain sequences in these groups, similar to the loss of activity for HPF30_0484 in Group 2, or could have been for another reason. We could not exclude the possibility that other unannotated RM systems were present, although by BLASTN [54] analysis with known Type I S and Type IIG genes did not find additional RM systems.

### Hemimethylated nonpalindromic methylation motifs

We identified 18 or fewer hemimethylated nonpalindromic methylation motifs that were not Type I-like but similar to the methylation motifs of Type III and subclasses of Type II RM systems [9], [55] (Table 6). *H. pylori* strains carry up to five loci for Type III M genes, each of which contains a TRD [19], [20]. One Type III TRD, in HPF16_0033 and HPF30_0034, was assigned as recognizing 5′-GGCAA . This is because this methylation motif was detected only in F16 and F30 among the five strains and because this TRD represents the only one TRD with such distribution. Many Type III M genes and Type IIG genes were truncated by mutations [13] so that we could not assign them hemimetylated nonpalindromic motifs. Only few of the TRD sequences identified in the apparently intact Type III M gene ORFs were shared by more than two strains used in the present work [13].

Four 7 bp methylation motifs were found in F30 (Table 6). These methylation motifs, 5′-CAAGWAG, 5′-CRTGHAG, 5′-CTNGNAG and 5′-CCDGNAG, were included in a single degenerated methylation motif, 5′-CNNGNAG. However, the other methylation motifs represented as 5′-CNNGNAG were rarely methylated. A single M gene might recognize these four sequences or four different M genes might recognize each sequence.

### Effect of a specificity gene on the methylome and transcriptome

As the first step to examine the biological significance of the variety in methylation specificity, we examined the effect of a Type I S gene, HPP12_0797, on the transcriptome (DRA accession no. DRA001073). We replaced this gene of strain P12 by a kanamycin-resistance gene. Its derivative with insertion of this gene downstream of the S gene was constructed for control. Loss of methylation at the recognition sequence deduced above, 5′-GAAN_8_TAG, was confirmed by comparison of their decoded methylomes (Table S2).

**Figure 6:**
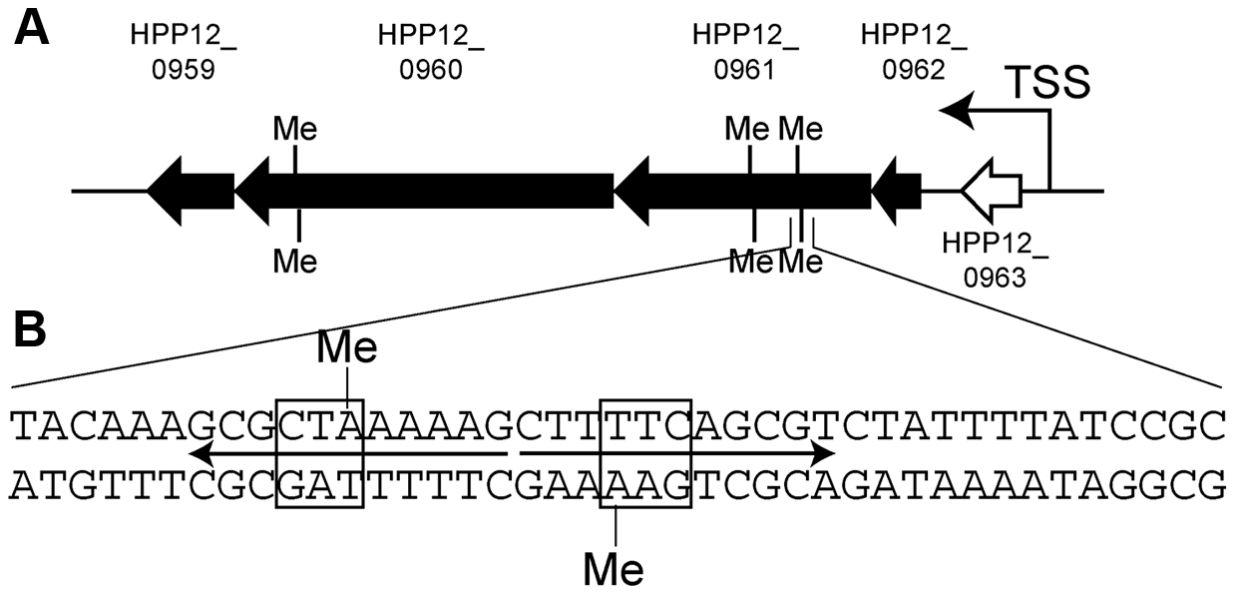
Gene cluster with transcripts decreased by specific Type I methylation. (A) Map. Black arrow, gene whose transcript was decreased by methylation; Black bar, position of methylation motif (5′-GAAN_8_TAG). TSS, transcription start site. (B) Sequence around a methylation site. Box, the half of the recognitioin sequence; arrows, palindrome; The leftmost bp is coordinate 1022181 of the P12 genome. Me, methyl group.

Transcriptomes were analyzed for differentially expressed genes (Table 7). The results were confirmed by quantitative real-time PCR. Transcripts of the S gene itself were absent in the knockout strain as expected. The gene cluster HPP12_0959 through HPP12_0962 (Figure 6A) showed increased transcript accumulation in the knockout strain. This cluster appears to be an operon whose transcription start site is upstream of an HPP12_0963 ortholog in another strain [56]. These results showed that methylation by Type I M and S gene products can significantly repress expression of genes.

**Table 7:** Transcriptome affected by knockout of a Type I specificity gene.

Gene (locus tag)	Product annotation	Read count in RNA-seq				p-value	qPCR^c^
		S−a		S+			(S−/S+)
		Exp. 1^b^	Exp. 2	Exp. 1	Exp. 2		
HPP12_0008	Chaperone and heat shock protein (GroEL)	22459	52845	25301	46382	0.05	(Control)
HPP12_0797	Type I R-M system S protein	1	0	55	32	4.42E-09	-d
HPP12_0959	ATP/GTP-binding protein	27	8	1	0	0.0013	3.1
HPP12_0960	ATP/GTP-binding protein	325	414	5	29	8.52E-10	2.3
HPP12_0961	Hypothetical protein	493	694	6	24	3.80E-13	48
HPP12_0962	Hypothetical protein	164	213	4	5	6.53E-14	25

a S-, HPYF1; S+, HPYF2. HPP12_0797 gene was knocked out.

b Read counts were normalized by the number of non-rRNA mapped reads.

c An average of two experiments.

d No specific amplification in S- strain.

Three copies of the recognition sequence for the S gene product were found in the body of those genes with significant expression changes (Figure 6B). The first of them was within a 22 bp palindromic sequence. We do not know whether this is a binding site for a symmetrical protein and we do not yet know the relationship, if any, between the long palindromic sequence and the transcriptional changes.

## Discussion

In this work, we analyzed the methylome of five *H. pylori* strains and two isogenic derivatives of one of the strains using SMRT sequencing technology. We also compared methylomes to infer recognition sequences of DNA methyltransferases. Large strain diversity was seen in the number of methylated bases and the repertoire of methylation motifs, as expected from the diversity in the number and sequences of specificity-determining genes. Examination of the distribution of methylated bases in genomes revealed hypermethylated and hypomethylated regions.

### Assignment of recognition sequences to specificity-determining genes

Assignment of methylation motifs to specificity-determining genes, that is Type I S, Type II M, and Type III M genes, is usually difficult for species with many of these genes. Most methylome analyses using SMRT sequencing used bacterial species with a small number of specificity-determining genes [36], [37]. Recognition sequence assignment in species and strains with a larger number of specificity-determining genes required cloning of each gene into well-defined *Escherichia coli* laboratory strains that lack DNA methyltransferase genes and methylation-specific nuclease genes [35]. The activities of specificity-determining genes can be different under intrinsic and cloned conditions and their expression level is critical for methylome analysis [34], [35].

*H. pylori* has a large number of specificity-determining genes. Because *H. pylori* strains also show large diversity in genome sequence [57], changes in methylation level and sequence specificity through subtle changes in amino acid sequence of specificity-determining genes was expected. To analyze the *H. pylori* methylome, strain differences in the repertoire of methylation motifs and in specificity-determining genes were used to assign methylation motifs to specificity-determining genes. This method also revealed a high diversity in target methylation level and sequence specificity between orthologous genes.

For Type I systems, methylation sequence assignment was carried out for two TRDs within each S gene product. Our analysis assigned a methylation motif to each Type I S gene and a half-recognition sequence to each TRD sequence (homology group). Of three groups of Type I S genes, two (Group 2 and Group 3) showed well-conserved methylation activity from apparently intact ORFs. These assignments combined together with the rules about the central repeats now allow prediction of target sequence of many S genes from their sequence.

For the other group of Type I S genes (Group 1) and Type IIG S genes, however, sufficient information on methylation motif was not obtained. One reason for the difficulty was the very low expression level of the Group 1 S gene, as revealed by transcriptome analysis of the P12-derived strains HPYF1 and HPYF2 (data not shown). Another reason was the absence of DoMo (Figure 1B) in Group 1. Some unassigned Type I-like methylation motifs were identified as candidates for recognition sequences of Type I S gene products, but these methylation motifs did not match the domain combinations for other groups of Type I S genes and Type IIG S genes. This result suggested that only a few combinations of TRD homology groups had methylation activity or that other unannotated S genes were present. We also cannot exclude the possibility of inactivation by simple mutation.

### Variation in methylation activity

Our detection of examples of strain-to-strain diversity in methylation activity by a specific methyltransferase suggested new mechanisms for inactivating a methylation system in addition to truncation by an insertion or deletion [20]. An untruncated gene that could not be assigned to a methylation motif in one strain could have an activity in another strain.

In addition to detecting complete inactivation of DNA methyltransferase genes with untruncated ORFs, we also observed intermediate methylation activity for some methylation motifs, such as methylation detection in 50–60% copies of methylation motif. This did not fit the hypothesis that the methylation level switches digitally between two states: 0% and 100%. Variation occurred even among members of the same ortholog group. A simple explanation for this variation is strain-specific mutations at residues important for activity. Indeed, many strain-specific amino acid changes were found within genes associated with the variable methylation levels. Another mechanism for the variation is competition for the recognition sequence among M or S genes with overlapping recognition sequences. We indeed detected many examples of recognition sequence overlap that had a substantial effect on the methylome. Methylation motifs that we did not detect by SMRT sequencing, such as those for 5-methylcytosine, are candidates for such competition because *H. pylori* has many strain-specific 5-methylcytosine methyltransferases [28].

### Microevolution in the sequence specificity of a methylation system

A related issue to the variation in methylation level is the subtle strain-to-strain variation in recognition sequence. Type I S gene TRDs in the same homology group might recognize different sequences in different strains: for example, 5′-C**C**A is recognized by one member of one TRD homology group in one strain and 5′-C**T**A is recognized by another member in another strain. Amino acid changes likely responsible for the changes were identified. Methylome comparisons of more strains might reveal more examples of such microevolution in sequence recognition, which would help our understanding of DNA sequence recognition by Type I S proteins.

Another type of microevolution we observed extended a recognition sequence from 5′-CCGG to 5′-CCGGH (H = A, T or C) for a Type II system. We do not yet know whether amino acid changes in the corresponding DNA methyltransferase or another factor was responsible for this change. We also noticed presence of several unassigned motifs within a strain that were similar to each other (Table 6CD). An example is a group of four sequences related to 5′-CNNGNAG.

These cases might be explained by intermediate stages in the switching of sequence specificity. Recent work on Type IIL restriction enzymes indicates their target specificity can be easily changed [9], [55].

### Biological significance

The results of this study revealed diversity in the methylome within *H. pylori* and demonstrated a built-in mechanism, DoMo, for generating this diversity. For Group 2 Type I S genes, we identified 5 active TRD homology groups. These groups generated diversity in S genes and their recognition sequences by allelic homologous recombination at TRDs and by DoMo. The copy number of central tandem repeats flanked by TRDs might change, resulting in variation in the number of Ns in a recognition sequence (Figure 1). For example, 10 TRD homology groups with 7 or 8 Ns at the center of methylation motif could generate 10×2×10×1/2 = 10^2^ structural variants of a single S gene. If one homology group correspsonds to one methylation motif, these structural variants correspond to sequence variants in the methylation motifs. Combined methylation sequence specificities could be even larger. Four such S loci would result in 10^2^×10^2^×10^2^×10^2^ = 10^8^ combined structural variants and a corresponding number of combined sequence specificities. Even greater diversity is possible when other types of sequence-specific DNA methyltransferases are considered.

What could be the biological significance of this enormous diversity? We earlier proposed, in an epigenetics-driven adaptive evolution model, that diverse methylomes serve as units of natural selection, with each unique gene experssion pattern and a unique set of phenotypes [14].

We observed that a Type I specificity gene affected the transcriptome. In eukaryotes, gene regulation frequently occurs through changes in protein binding affinity, for example of transcription factors, that is caused by methylation near promoters [1], [58]. Recent work shows that methylations within gene body might also regulate gene expression [6]. In prokaryotes, gene expression changes resulting from methylation distribution have been well studied [4], [20], [59]. Methylation of 5′-GATC, which was found here partly responsible for hypermethylation of an RNA polymerase gene, is important for gene expression regulation. Further work is necessary to elucidate the biological significance of the methylome data from this study.

We earlier proposed the hypothesis that specificity changes in methyltransferases might lead to changes in phenotype [14]. These changes might not only detract but also might have potential to enable adaptive evolution. Under this hypothesis, the roles of changes in methylation specificity could be similar to the roles of genome rearrangements in adaptive evolution, for example, the antigenic variation that results from gene conversion to adapt to host immunity [60]. We need to learn more about methylation specificity changes and their effects, if any, on phenotypes and genotypes to evaluate the functions of these changes.

In this work, we provided evidence that a bacterial species has an enormous diversity in methylome status through various mechanisms including point mutations and DoMo (movement of target recognition domain sequences between sites within a gene). Deletion of a methylation specificity-determining gene affected the transcriptome. These findings are consistent with our hypothesis that methylome changes might lead to changes in cell physiology through transcriptome changes, and might contribute to adaptive evolution. Epigenetic changes in DNA methylation might be a potential source of variation for adaptive evolution, similar to DNA sequence changes.

During the reviewing process of this manuscript, a paper decoding methylome of two other *H. pylori* strains appeared [39]. We have not noticed any inconsistency between these two works. In particular, their assignments of Type I S genes to the full target sequences and our assignments of the TRDs within S genes to the half target sequences are consistent.

## Materials and Methods

### Strains

*H. pylori* strain P12 [40] was kindly provided by Rainer Haas (Ludwig-Maximilians-University of Munich, Germany). *H. pylori* strains F16, F30, F32 and F57 were previously described [17]. According to multilocus sequence typing based on seven housekeeping genes, P12 belongs to the hpEurope group and F16, F30, F32 and F57 belong to the hspEAsia group [17].

### *H. pylori* culture and genome preparation

Strains were inoculated from 50% glycerol stocks onto tripticase soy agar (TSA)-II/5% sheep blood plates (Becton Dickinson, NJ) and incubated under microaerobic conditions (O_2_, 5%; CO_2_, 15%; N_2_, 80%) at 37°C for 3 days. Colonies were collected by resuspending in 1 ml Brucella (Becton Dickinson, NJ) broth, and transferred to 99 ml of Brucella broth with 10% fetal calf serum. After growth for one day under microaerobic conditions, cells were centrifuged at 8000×*g* for 5 min and the supernatant discarded. Genomic DNA was extracted from the pellet by a protease/phenol method as described elsewhere [17] and resuspended in 300 μl of TE buffer (10 mM Tris HCl, pH 7.8).

### Mutant strain construction

A region covering the HPP12_0797 ORF and 1 kb flanking sequences on both sides was amplified by PCR with KOD FX Neo (TOYOBO, Japan) using primers P12_group2S_EcoRI_for (GGGGAATTCGGAATTACAAGGGTTTCAGCATTCAGCC) and P12_group2S_BamHI_rev (GGGGGATCCGCTTACCCAAGCTAAAAGCATCGC). Amplified fragments were cleaved with EcoRI and BamHI, followed by ligation with Ligation high Ver.2 (TOYOBO, Japan) to pBR322 cleaved with the same enzymes. Ligation products were transformed into *E. coli* DH10B competent cells by electroporation, resulting in plasmid pYF166.

For replacement of HPP12_0797 on pYF166 with a kanamycin-resistance gene, pYF166 other than HPP12_0797 ORF region was amplified using primers P12_group2S_sub_ClaI_for (GGGATCGATGCCCTTCTTCTAAATGGCTAATG) and P12_group2S_sub_KpnI_rev (GGGGGTACCCAAAATACCCCCCTATCCCC). For preparation of a control strain with a kanamycin-resistance gene at the downstream of HPP12_0797, almost whole the pYF166 was amplified using primers P12_group2S_sub_ClaI_confrol_for (GGGATCGATCCCCCTTAACCCCCAACTAG) and P12_group2S_sub_KpnI_rev.

The kanamycin-resistance gene was amplified by PCR from pHel3 [61], kindly provided by Rainer Haas (Ludwig-Maximilians-University of Munich, Germany), using primers P3_ClaI (GGGATCGATAAAATTGGAACCGGTACGCTTA) and P4_KpnI (GGGGGTACCAGACATCTAAATCTAGGTAC) [62]. Amplified fragments with the kanamycin-resistance gene were cleaved with ClaI and KpnI and ligated with Ligation high Ver.2 (TOYOBO, Japan) to fragments from pYF166 (described above) cleaved with the same enzymes. Ligation products were transformed into DH10B by electroporation to obtain pYF171 (knockout) and pYF173 (control).

For transformation into *H. pylori* P12, inserts in pYF171 and pYF173 were amplified by PCR with primers P12_group2S_EcoRI_for and P12_group2S_BamHI_rev. A P12 culture was prepared as described above and 1 μg of amplified DNA fragment was added. After one day growth under microaerobic conditions at 37°C, 200 μl culture was plated on TSA-II/5% sheep blood plates with 8 mg/L kanamycin and incubated at 37°C for 3 days under microaerobic conditions. Single-colony isolation was carried out under the same conditions, resulting in the HPP12_0797-knockout strain, HPYF1, and the control strain, HPYF2. Cultures were prepared as described above and stored at -80°C as 50% glycerol stocks.

### SMRT sequencing

Genomic DNA samples were sheared to ∼500 bp using a S2 Focused-ultrasonicator (Covaris, MA). SMRT bell libraries for SMRT sequencing were prepared with DNA Template Prep Kit 2.0 (Pacific Biosciences, CA) (250 bp <3 kb). SMRT sequencing was performed using a DNA Sequencing Kit 2.0 with C2 polymerase (Pacific Biosciences, CA), following standard instructions for a PacBio RS (Pacific Biosciences, CA). Two biological replicates were performed for each strain. The read depth was approximately ×100 (Table S2).

SMRT sequencing data were analyzed by the RS_Modification_and_Motif_Analysis.1 protocol in SMRT Analysis version 1.4.0 through the SMRT Portal. In brief, reads were mapped to the genome sequences (Accession numbers: P12, NC_011498; F16, AP011940; F30 chromosome, AP011941; F30 plasmid, AP011942; F32 chromosome, AP011943; F32 plasmid, AP011944; F57, AP011945). Interpulse durations were measured for all nucleotide positions in the genomes and compared with expected durations in a kinetic model of the polymerase [63] for significant associations.

To analyze methylation distribution, the number of methylated bases in a 1 kb window was counted with sliding by 500 bp for each strand.

All 20 bp sequences upstream and downstream of a methylated nucleotide that were not in a methylation motif detected by the above protocol were collected and searched for methylation motifs by MEME-ChIP [64]. Score thresholds were chosen to fulfill the condition that methylation positions without detectable methylation motifs after MEME-ChIP analysis were less than 5% of the number of detected methylated positions. A methylation motif was assumed to be methylated if more than 20% of copies of the methylation motif in the genome were detected as methylated in both biological replicates.

### Strand-specific RNA-seq

HPYF1 and HPYF2 were grown to OD_600_ of 0.3–0.4 and cell pellets were prepared as described above. Whole RNA was extracted by PureLink RNA Mini Kit (Life Technologies, MD). RNA samples were prepared with mRNA-Seq Sample Prep Kit (Illumina, CA) for construction of libraries for strand-specific RNA-seq. Libraries were sequenced by HiSeq 2000 (Illumina, CA).

Resulting sequences were mapped to protein coding sequences in the P12 genome (Accession number: NC_011498). Mapped read counts for each coding sequence were compared by the DESeq package to detect significant differences in expressed genes using a threshold of P<0.001 [65].

Quantitative real-time PCR used KAPA SYBR FAST One-Step qRT-PCR Kit ABI Prism (KAPA Biosystems, MA). Each sample was analyzed as triplicate technical replicates. HPP12_0008, encoding GroEL, was used as an internal control [56]. An Applied Biosystems 7300 Real-Time PCR System (Life Technologies, MD) was used for detection and analysis. Primers are in Table S4.

## Supporting Information

Figure S1 Methylome decoding in five *H. pylori* strains. (A) Strain P12. (B) F16. (C) F30. (D) F32. (E) F57. Interpulse duration scores were plotted for each nucleotide in the genomes, clockwise (5′ to 3′) (outer) or counterclockwise (5′ to 3′) (inner) using Circos [66]. Smaller ticks in the inner circle, 10 kb; larger ticks, 100 kb; black bar, coordinate zero.
(PDF)

Figure S2 Distribution of methylated bases on each strand of each strain. (A) Strain P12. (B) F16. (C) F30. (D) F32. (E) F57.
(PDF)

Figure S3 Distribution of methylated sites in two hypermethylated genes (*rpoB* and *groEL*). (A) Strain P12 *rpoB*. (B) F16 *rpoB*. (C) F30 *rpoB*. (D) F32 *rpoB*. (E) F57 *rpoB*. (F) P12 *groEL*. (G) F16 *groEL*. (H) F30 *groEL*. (I) F32 *groEL*. (J) F57 *groEL*. Strain name is followed by gene name.
(PDF)

Figure S4 Sequence alignments of genes determining target sequence specificity. (A) HPP12_0044 homologs. (B) HPP12_0262 homologs. (C) HPP12_0908 homologs. (D) HPP12_0259 homologs. (E) HPP12_1523 homologs. Type, gene name, and recognition sequence are indicated. Amino acids identical in all strains are shaded. Roman numerals indicate amino acid sequence motifs conserved among DNA methyltranferases [53].
(PDF)

Table S1 Overview of outputs in the strains.
(XLSX)

Table S2 Methylome results.
(XLSX)

Table S3 Unassigned methylation motifs.
(XLSX)

Table S4 Primers for qPCR.
(XLSX)
