# Supplementary figures and images for: Methylome Diversification through Changes in DNA Methyltransferase Sequence Specificity
Source: PLoS Genet. 2014 Apr 10;10(4):e1004272. doi: 10.1371/journal.pgen.1004272 (PMC3983042; doi:10.1371/journal.pgen.1004272)

A

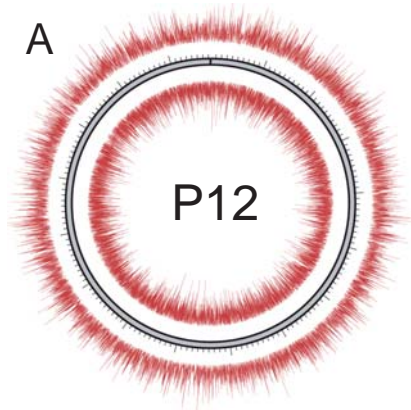

B

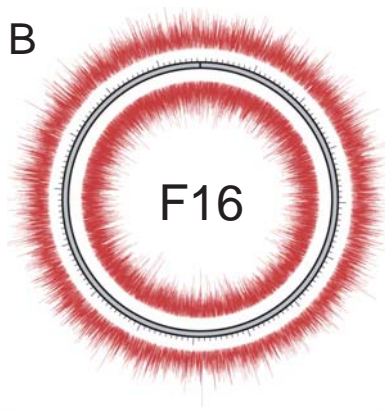

C

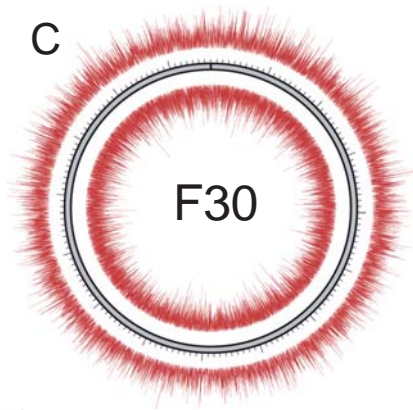

D

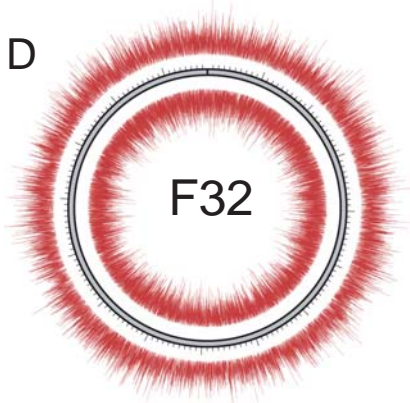

E

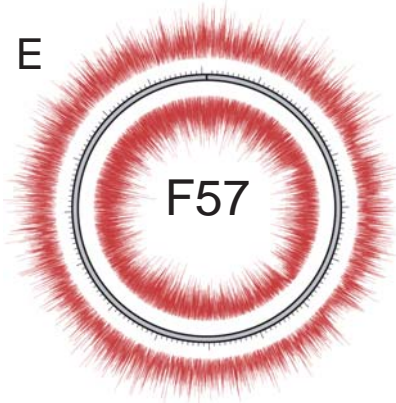

Supplement: Figure S1 — Methylome decoding in five H. pylori strains. (A) Strain P12. (B) F16. (C) F30. (D) F32. (E) F57. Interpulse duration scores were plotted for each nucleotide in the genomes, clockwise (5′ to 3′) (outer) or counterclockwise (5′ to 3′) (inner) using Circos [66]. Smaller ticks in the inner circle, 10 kb; larger ticks, 100 kb; black bar, coordinate zero. (PDF) [file pgen.1004272.s001.pdf]

**A P12**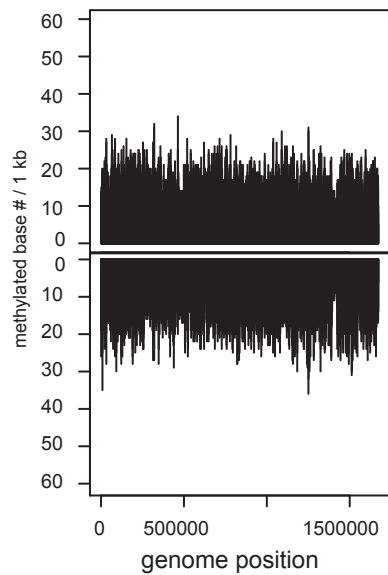**B F16**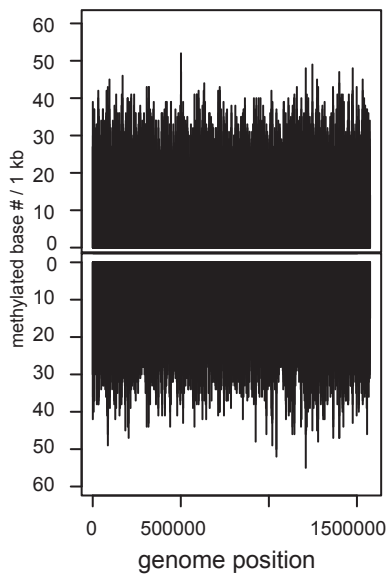**C F30**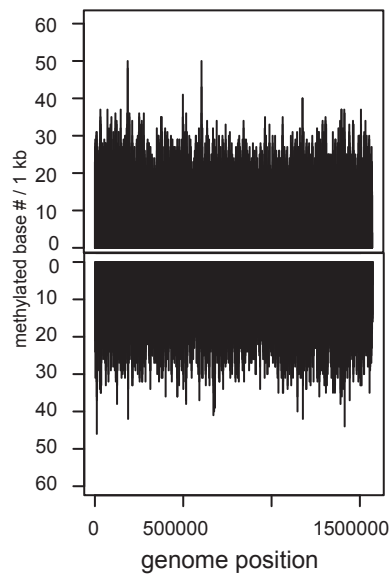**D F32**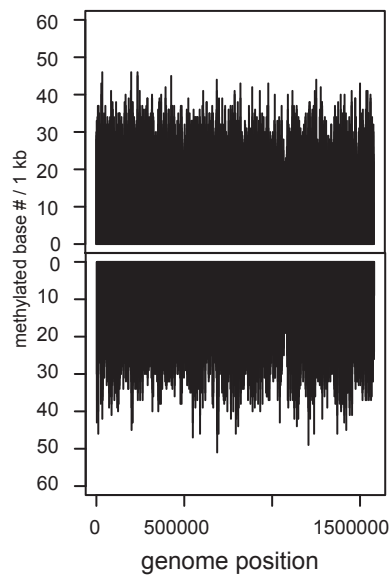**E F57**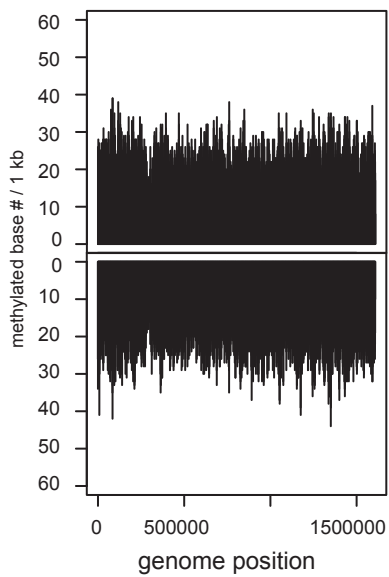

Supplement: Figure S2 — Distribution of methylated bases on each strand of each strain. (A) Strain P12. (B) F16. (C) F30. (D) F32. (E) F57. (PDF) [file pgen.1004272.s002.pdf]
