# Supplementary material for: Methylome Diversification through Changes in DNA Methyltransferase Sequence Specificity
Source: PLoS Genet. 2014 Apr 10;10(4):e1004272. doi: 10.1371/journal.pgen.1004272 (PMC3983042; doi:10.1371/journal.pgen.1004272)
Supplement: Figure S3 — Distribution of methylated sites in two hypermethylated genes (rpoB and groEL). (A) Strain P12 rpoB. (B) F16 rpoB. (C) F30 rpoB. (D) F32 rpoB. (E) F57 rpoB. (F) P12 groEL. (G) F16 groEL. (H) F30 groEL. (I) F32 groEL. (J) F57 groEL. Strain name is followed by gene name. (PDF) [file pgen.1004272.s003.pdf]

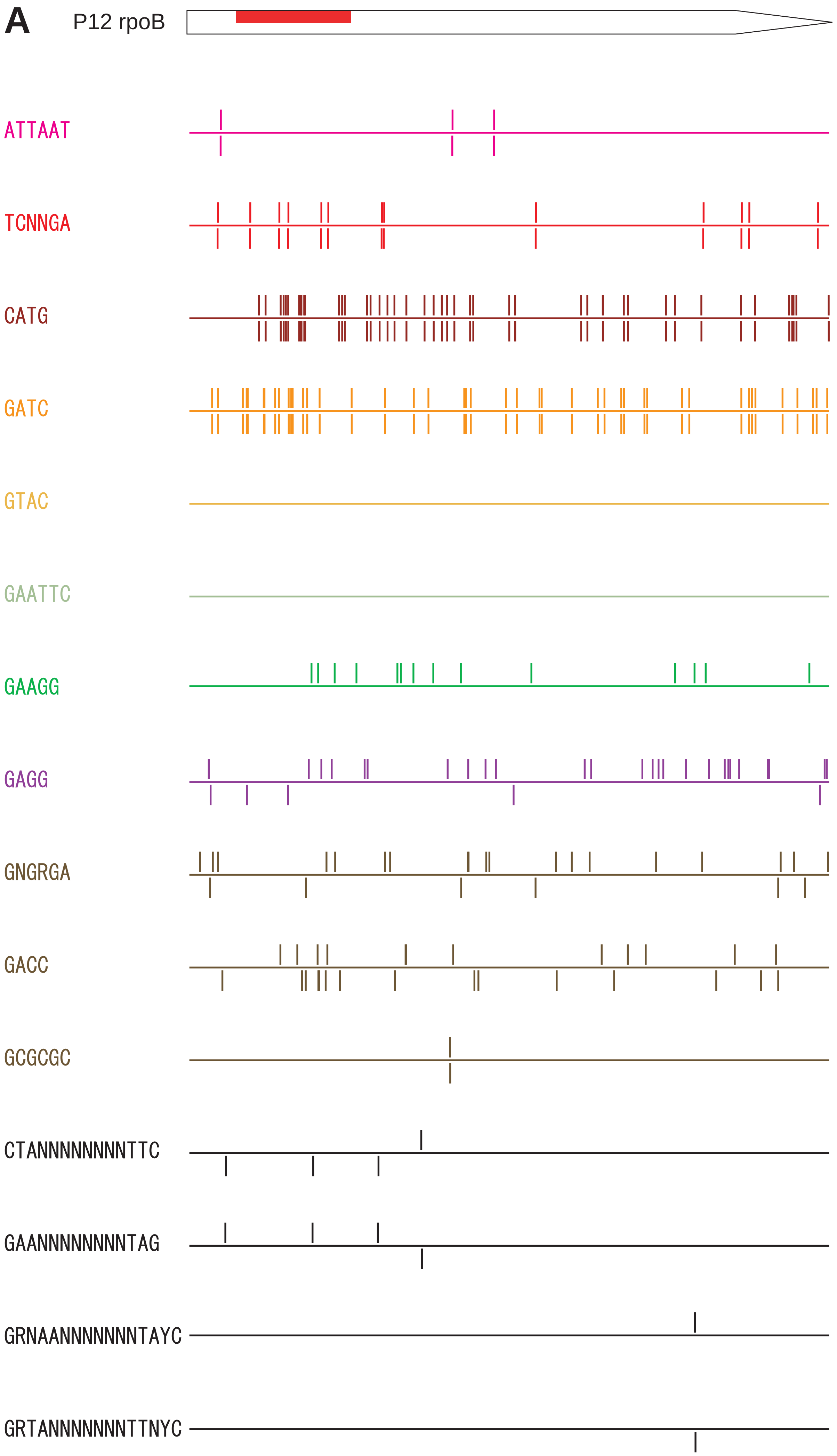
A
P12 rpoB
ATTAAT
TCNNGA
CATG
GATC
GTAC
GAATTC
GAAGG
GAGG
GNGRGA
GACC
GCGCGC
CTANNNNNNNNTTC
GAANNNNNNNNTAG
GRNAANNNNNNNTAYC
GRTANNNNNNNTTNYC

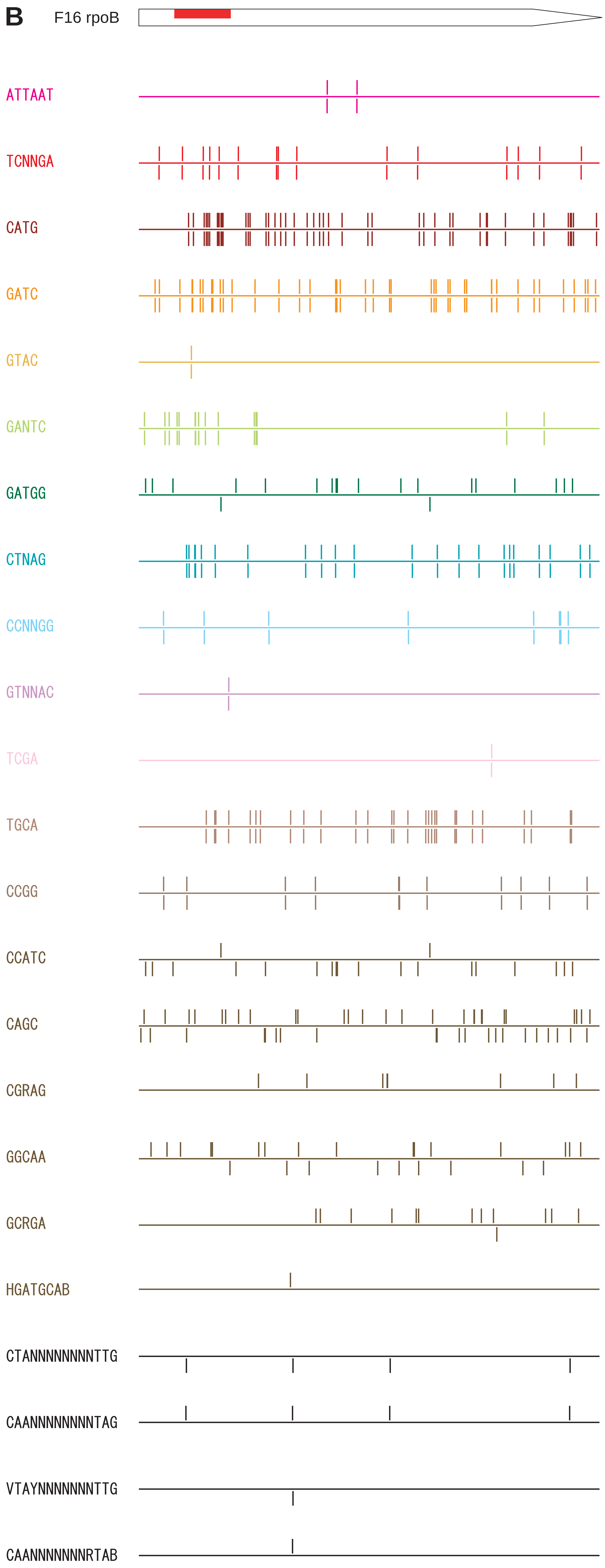
B
F16 rpoB
ATTAAT
TCNNGA
CATG
GATC
GTAC
GANTC
GATGG
CTNAG
CCNNGG
GTNNAC
TCGA
TGCA
CCGG
CCATC
CAGC
CGRAG
GGCAA
GCRGA
HGATGCAB
CTANNNNNNNNTTG
CAANNNNNNNNTAG
VTAYNNNNNNNTTG
CAANNNNNNNRTAB

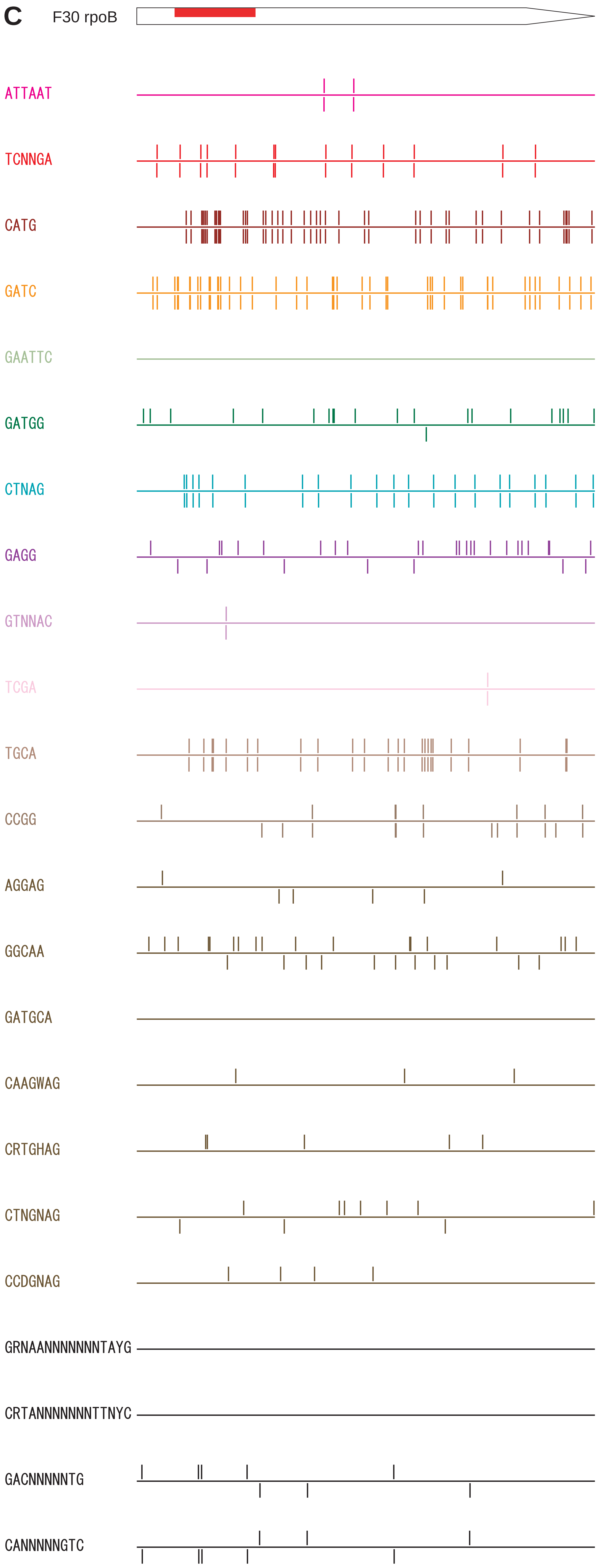
C
F30 rpoB
ATTAAT
TCNNGA
CATG
GATC
GAATTC
GATGG
CTNAG
GAGG
GTNNAC
TCGA
TGCA
CCGG
AGGAG
GGCAA
GATGCA
CAAGWAG
CRTGHAG
CTNGNAG
CCDGNAG
GRNAANNNNNNNTAYG
CRTANNNNNNNTTNYC
GACNNNNNTG
CANNNNNGTC

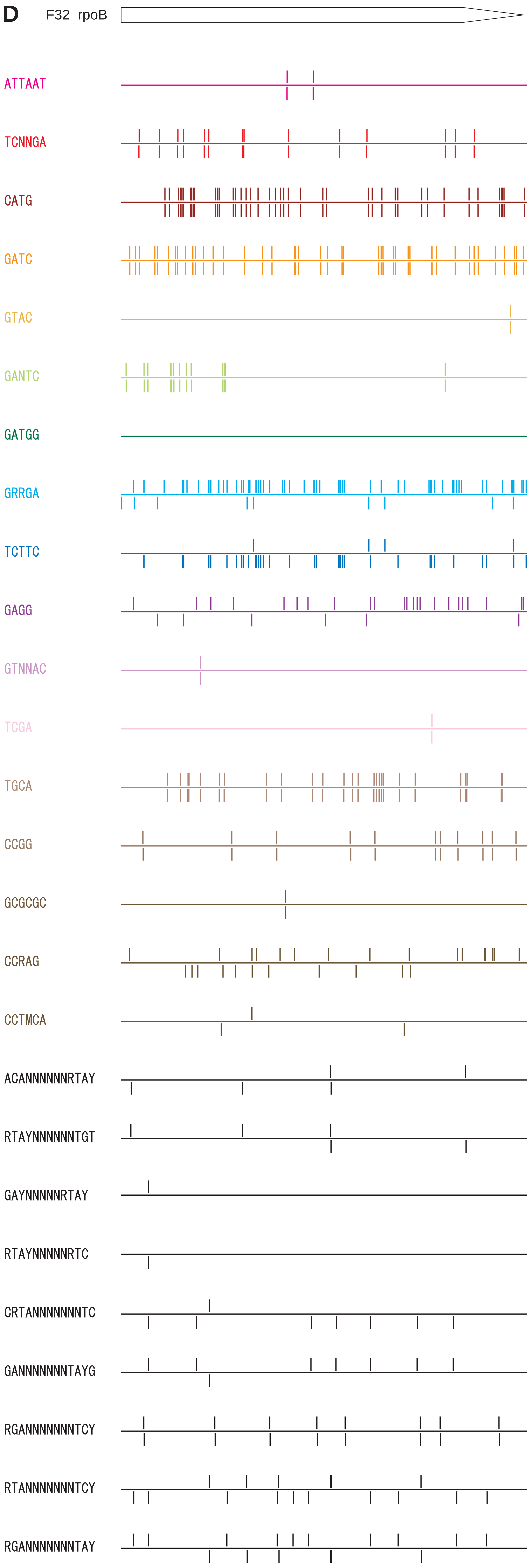
D
F32 rpoB
ATTAAT
TCNNGA
CATG
GATC
GTAC
GANTC
GATGG
GRRGA
TCTTC
GAGG
GTNNAC
TCGA
TGCA
CCGG
GCGCGC
CCRAG
CCTMCA
ACANNNNNNRTAY
RTAYNNNNNNTGT
GAYNNNNNRTAY
RTAYNNNNNRTC
CRTANNNNNNNTC
GANNNNNNNTAYG
RGANNNNNNNTCY
RTANNNNNNNTCY
RGANNNNNNNTAY

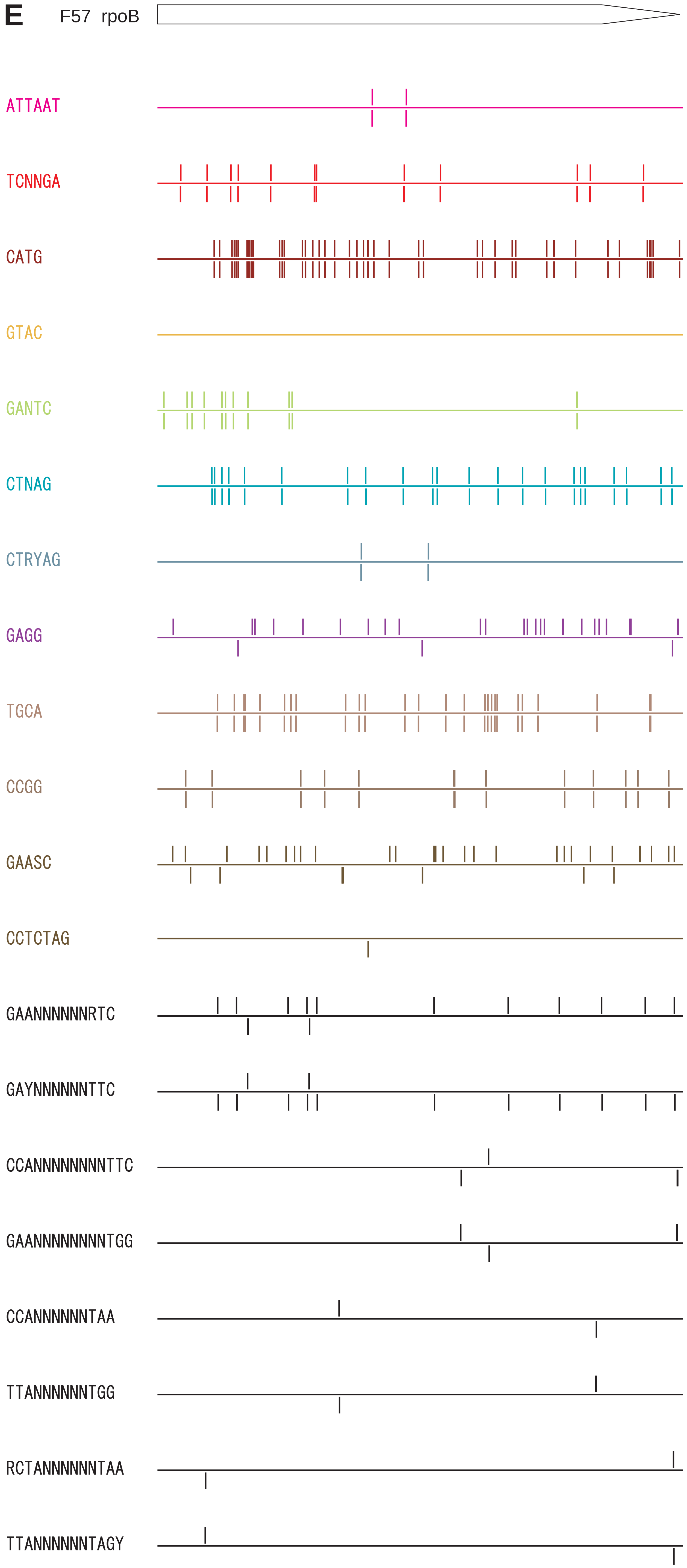
E
F57 rpoB
ATTAAT
TCNNGA
CATG
GTAC
GANTC
CTNAG
CTRYAG
GAGG
TGCA
CCGG
GAASC
CCTCTAG
GAANNNNNNRTC
GAYNNNNNNTTC
CCANNNNNNNNTTC
GAANNNNNNNNTGG
CCANNNNNNTAA
TTANNNNNNTGG
RCTANNNNNNTAA
TTANNNNNNTAGY

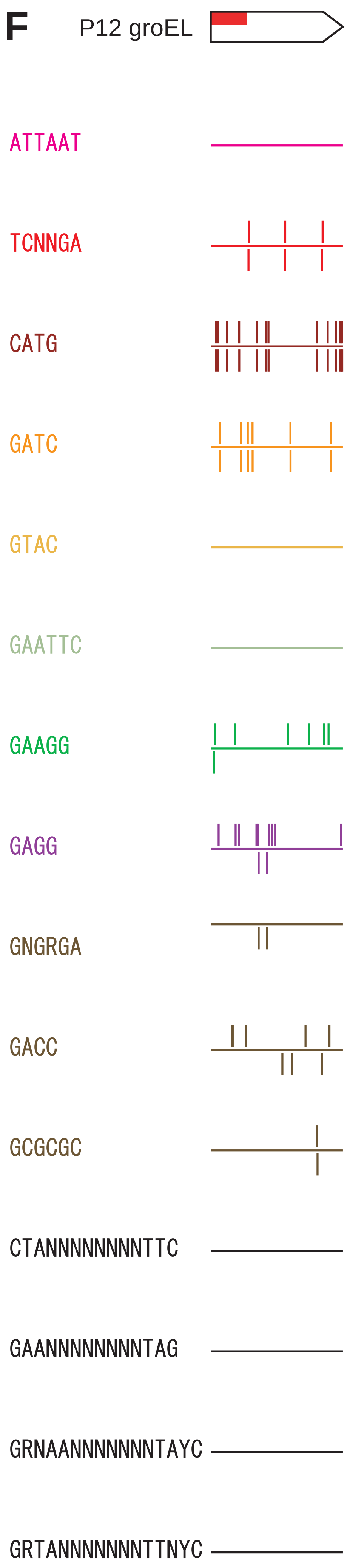
F
P12 groEL
ATTAAT
TCNNGA
CATG
GATC
GTAC
GAATTC
GAAGG
GAGG
GNGRGA
GACC
GCGCGC
CTANNNNNNNNTTC
GAANNNNNNNNTAG
GRNAANNNNNNNTAYC
GRTANNNNNNNTTNYC

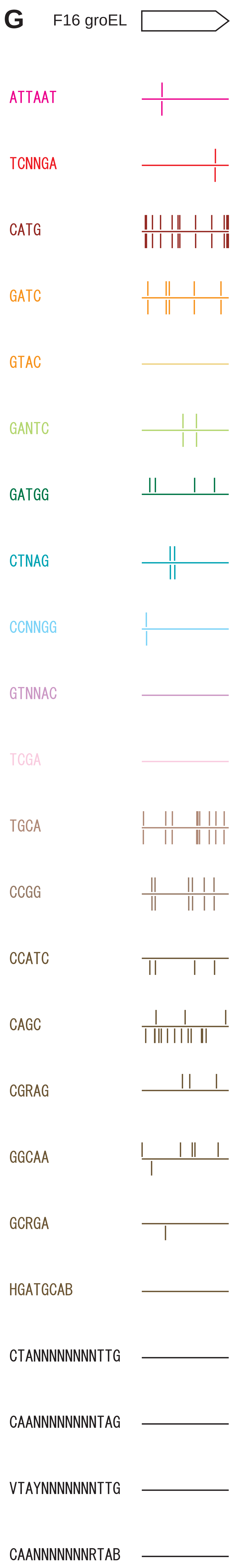
G
F16 groEL
ATTAAT
TCNNGA
CATG
GATC
GTAC
GANTC
GATGG
CTNAG
CCNNGG
GTNNAC
TCGA
TGCA
CCGG
CCATC
CAGC
CGRAG
GGCAA
GCRGA
HGATGCAB
CTANNNNNNNNTTG
CAANNNNNNNNTAG
VTAYNNNNNNNTTG
CAANNNNNNNRTAB

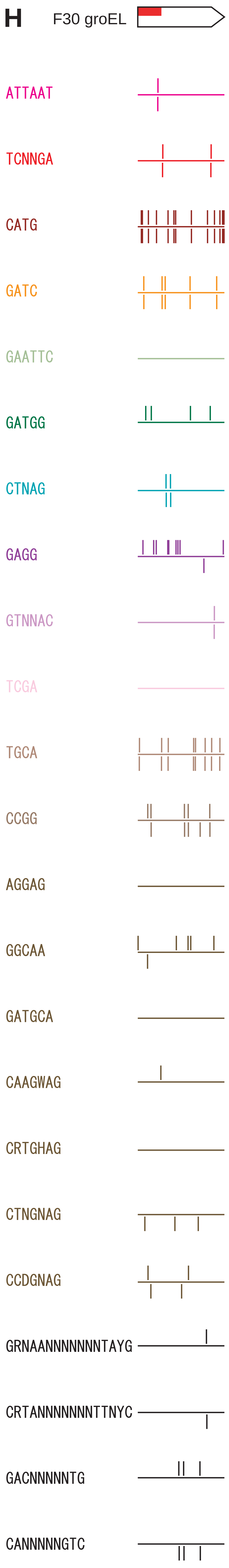
H
F30 groEL
ATTAAT
TCNNGA
CATG
GATC
GAATTC
GATGG
CTNAG
GAGG
GTNNAC
TCGA
TGCA
CCGG
AGGAG
GGCAA
GATGCA
CAAGWAG
CRTGHAG
CTNGNAG
CCDGNAG
GRNAANNNNNNNTAYG
CRTANNNNNNNTTNYC
GACNNNNNTG
CANNNNNGTC

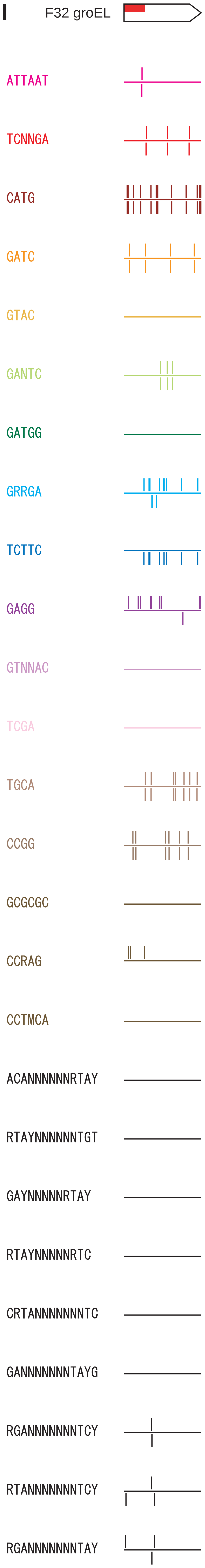
F32 groEL
ATTAAT
TCNNGA
CATG
GATC
GTAC
GANTC
GATGG
GRRGA
TCTTC
GAGG
GTNNAC
TCGA
TGCA
CCGG
GCGCGC
CCRAG
CCTMCA
ACANNNNNNRTAY
RTAYNNNNNNTGT
GAYNNNNNRTAY
RTAYNNNNNRTC
CRTANNNNNNNTC
GANNNNNNTAYG
RGANNNNNNNTCY
RTANNNNNNNTCY
RGANNNNNNNTAY

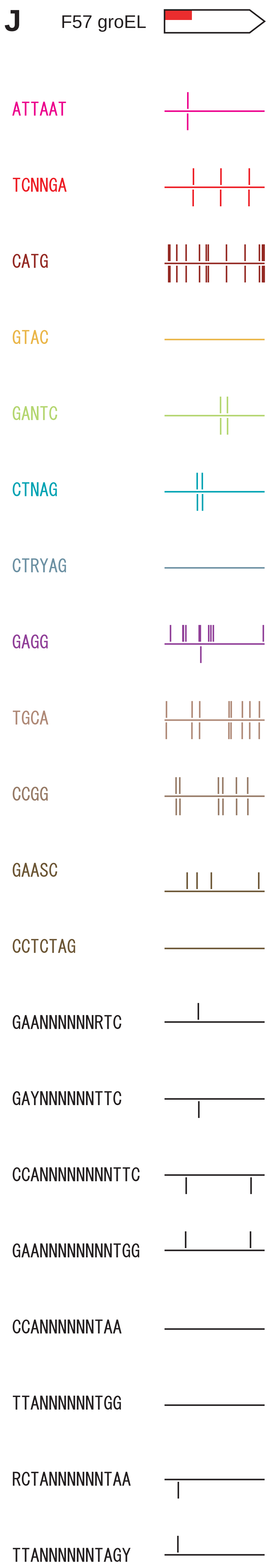
J
F57 groEL
ATTAAT
TCNNGA
CATG
GTAC
GANTC
CTNAG
CTRYAG
GAGG
TGCA
CCGG
GAASC
CCTCTAG
GAANNNNNNRTC
GAYNNNNNNTTC
CCANNNNNNNNTTC
GAANNNNNNNNTGG
CCANNNNNNTAA
TTANNNNNNTGG
RCTANNNNNNTAA
TTANNNNNNTAGY
