# Supplementary material for: Methylome Diversification through Changes in DNA Methyltransferase Sequence Specificity
Source: PLoS Genet. 2014 Apr 10;10(4):e1004272. doi: 10.1371/journal.pgen.1004272 (PMC3983042; doi:10.1371/journal.pgen.1004272)
Supplement: Figure S4 — Sequence alignments of genes determining target sequence specificity. (A) HPP12_0044 homologs. (B) HPP12_0262 homologs. (C) HPP12_0908 homologs. (D) HPP12_0259 homologs. (E) HPP12_1523 homologs. Type, gene name, and recognition sequence are indicated. Amino acids identical in all strains are shaded. Roman numerals indicate amino acid sequence motifs conserved among DNA methyltranferases [53]. (PDF) [file pgen.1004272.s004.pdf]

A (Type II, beta, 5'-GAGG)

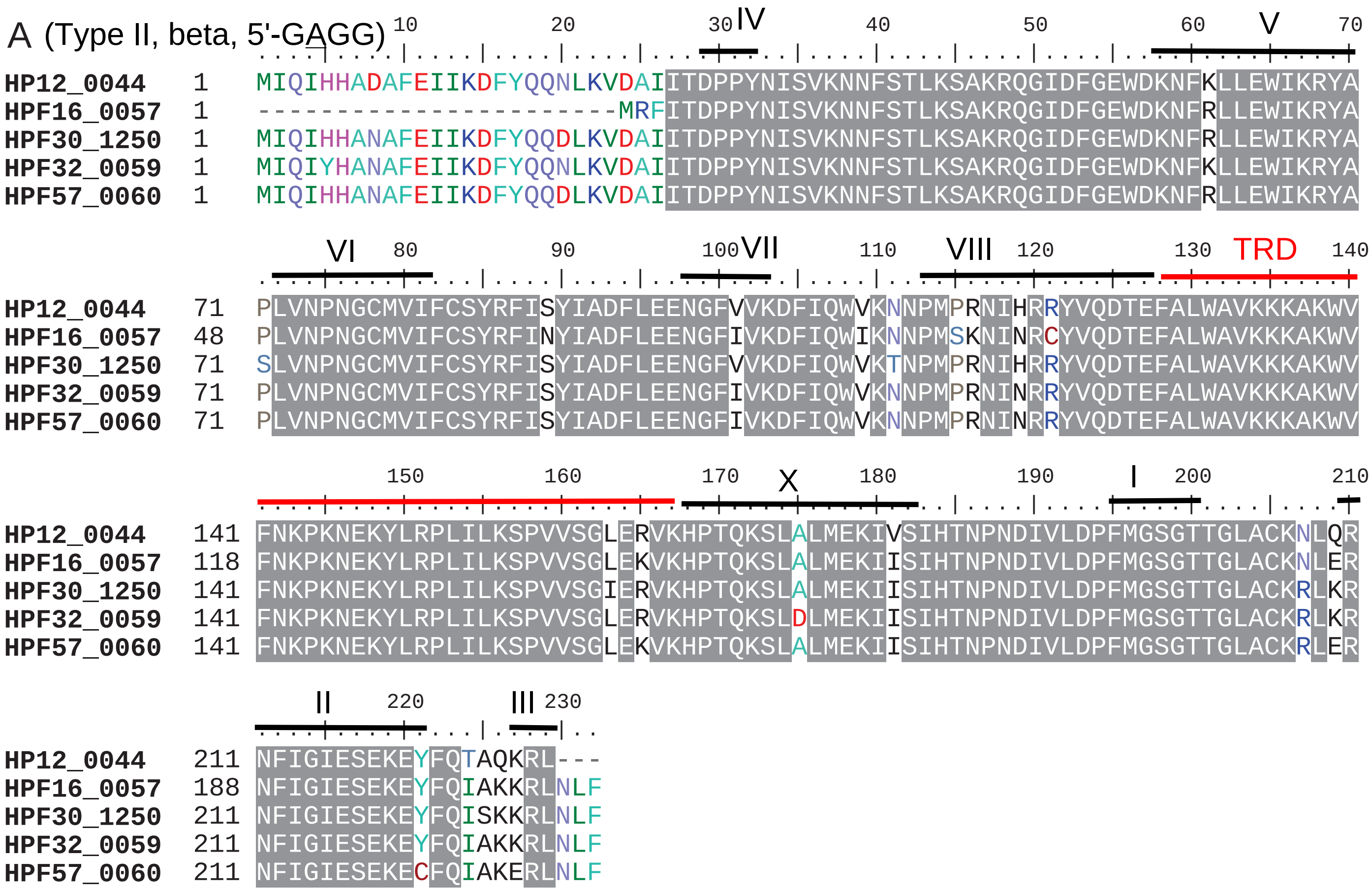

B (Type II, beta, 5'-CCGG)

```
                                  10        20        30   IV   40        50     V  60        70
                              ....|....|....|....|....|....|....|....|....|....|....|....|....|....|
HPP12_0262   1   MKPYFSLEKLDLYHGDASVLETFEKGFYDLCVTSPPYNLSIEYQGSNDFRAYDDYLNWCKNWLKNCYFWG
HPF16_0270   1   MKPYFSLEKLDLYHGDVSVLETFEKGFYDLCVTSPPYNLSIEYQGSNDFRVYDDYLNWCKNWLKNCYFWG
HPF30_1033   1   MKPYFSLEKLDLYHGDASVLETFEKGFYDLCITSPPYNLSIEYQGSNDFRAYDDYLNWCKNWLKNCYFWG
HPF32_0272   1   MKPYFSLEKLDLYHGDASVLKTFEKGFYDLCITSPPYNLSIEYQGSNDFRAYDDYLNWCKNWLKNCYFWG
HPF57_0316   1   MKPYFSLEKLDLYHGDVSVLETFEKGFYDLCITSPPYNLSIEYQGSNDFRAYDDYLNWCKNWLKNCYFWG

                        VI   80     VII  90        100       110 VIII      120       130       140
                              ....|....|....|....|....|....|....|....|....|....|....|....|....|....|
HPP12_0262   71  KEQARLCLNVPLDTNKHGKQSLGADIIAVAKECGWKYQNTIIWNESNISRRTAWGSWLQASTPYAIAPVE
HPF16_0270   71  KEQARLCLNVPLDTNKHGKQSLGADIIAIAKECGWKYQNTIIWNESNISRRTAWGSWLQASAPYAIAPVE
HPF30_1033   71  KEQARLCLNVPLDTNKHGKQSLGADMIAIAKECGWKYQNTIIWNESNISRRTAWGSWLQASASYAIAPVE
HPF32_0272   71  KEQARLCLNVPLDTNKHGKQSLGADMIAIAKECGWKYQNTIIWNESNISRRTAWGSWLQASAPYAIAPVE
HPF57_0316   71  KEQARLCLNVPLDTNKHGKQSLGADIIAIAKECGWKYQNTIIWNESNISRRTAWGSWLQASAPYAIAPVE

                                       150   TRD   160       170       180   X   190       200       210
                              ....|....|....|....|....|....|....|....|....|....|....|....|....|....|
HPP12_0262   141 LIVVFYKNEYKRQKQTSTISKEEFLLYTNGLWSFSGESKKCLKHPAPFPRELPRRCIQLFSFLEDTIFDP
HPF16_0270   141 LIVIFYKNEYKRQKQTSTISKEEFLLYTNGLWSFSGESKKRLKHPAPFPRELPRRCIKLFSFLEDTIFDP
HPF30_1033   141 LIVVFYKNAYKRQKQTSTISKEEFLLYTNGLWSFSGESKKRLKHPAPFPRELPRRCIKLFSFLEDTIFDP
HPF32_0272   141 LIVVFYKNEYKRQKQTSTISKEEFLLYTNGLWSFSGESKKRLKHPAPFPRELPRRCIKLFSFLEDTIFDP
HPF57_0316   141 LIVVFYKNEYKRQKQTSTISKEEFLLYTNGLWSFSGESKKRLKHPAPFPRELPRRCIKLFSFLEDTIFDP

                   I        220       230II      240   III 250
                              ....|....|....|....|....|....|....|....|...
HPP12_0262   211 FSGSGTTILEANALGRFSVGLEIEKEYCELSKKRILESLSLV*
HPF16_0270   211 FSGSGTTILEANALGRFSVGLEIEKEYCELSKKRILESLSLVR
HPF30_1033   211 FSGSGTTILEANALGRFSVGLEIEKEYCELSKKRILESLSLV*
HPF32_0272   211 FSGSGTTILEANALGRFSVGLEIEKEYCELSKKRILESLSLV*
HPF57_0316   211 FSGSGTTILEANALGRFSVGLEIEKEYCKLSKKRILESLSLV*
```

C (Type II, gamma, 5'-GTNNAC)

```
                    10        20        30        40        50        60        70
                         X                          I                 II
HPP12_0908   1   LENFLNNLDIKTLGQVFTPKNIVDFMLTLKHNQGSVLEPSAGDGSFLKRLKKAVGIEIDPKICPKNALCM
HPF16_0891   1   LEGFLYQLDIKTLGQVFTPKNIVDFMLTLKQNHGSVLEPSTGDGSFLKHLKKAVGIEIDPKICPKNALCM
HPF30_0429   1   LENFLNNLDIKTLGQVFTPKKIVDFMLTLKQNQGSVLEPSAGDGSFLKRLKKAVGIEIDPKICPKNALCM
HPF32_0444   1   LEGFLYQLDIKTLGQVFTPKNIVDFMLTLKQNHGSVLEPSAGDGSFLKRLKKAVGIEIDPKICPKNALCM
HPF57_0920   1   LEGFLYQLDIKTLGQVFTPKNIVDFMLTLKQNHGSVLEPSAGDGSFLKRLKKAVGIEIDPKICPKNALCM

                 III      80        90       100       110       120       130       140
                               IV                          V                VI
HPP12_0908  71   DFFDYPLENQFDTIIGNPPYVKHKDIAPSAKEKLHYSLFDERSNLYLFFIEKAIKHLKPQGELIFITPRD
HPF16_0891  71   DFFDYPLENQFDTIIGNPPYVKHKDIAPSTKEKLHYSLFDERSNLYLFFIEKAIKHLKPKGELIFITPRD
HPF30_0429  71   DFFDYPLENQFDTIIGNPPYVKHKDIAPSTKEKLHYSLFDERSNLYLFFIEKAIKHLKPKGELIFITPRD
HPF32_0444  71   DFFDYPLENQFDTIIGNPPYVKHKDIAPSTKEKLHYSLFDERSNLYLFFIEKAIKHLKPKGELIFITPRD
HPF57_0920  71   DFFDYPLENQFDTIIGNPPYVKHKDIAPSAKEKLNYSLFDERSNLYLFFIEKAIKHLKPKGELIFITPRD

                          150 VII       160       170 VIII    180       190       200       210
HPP12_0908 141   FLKSTSSVKLNEWIYKEGTITHFFELGDQKIFPNAMPNCVIFRFCKGNFSRIANDGLQFLCKKGILYFLN
HPF16_0891 141   FLKSTSSVKLNELIYKEGTITHFFELGDQKVFPNAMPNCVIFRFCKGNFSRITNDCLQFLCKKGILYFLN
HPF30_0429 141   FLKSTSSVKLNELIYKEGTITHFFELGDQKVFPNAMPNCVIFRFCKGNFSRITNDCLQFLCKKGILYFLN
HPF32_0444 141   FLKSTSSVKLNEWIYKEGTITHFFELGDQKVFPNAMPNCVIFRFCKGNFSRITNDGLQFLCKKGILYFLN
HPF57_0920 141   FLKSTSSVKLNEWIYKEGTITHFFELGDQKVFPNAMPNCVIFRFCKGNFSRITNDCLQFLCKKGILYFLN

                          220       230       240  TRD  250       260       270       280
HPP12_0908 211   QSYTQKLSEVFKVKVGAVSGCDKIFKNEKYGNLEFVTSITKRTNVLEKMVFVNKPNDYLLQHKDSLMQRK
HPF16_0891 211   QSYTQKLSEVFKVKVGAVSGCDKIFKNEKYGNLEFVTSITKRTNVLEKMVFVNKPNDYLLQHKDSLMQRK
HPF30_0429 211   QSYTQKLSEIFKVKVGAVSGCDKIFKNEKYGNLEFVTSITKRTNVLEKMVFVNEPNDYLLQHKDSLMQRK
HPF32_0444 211   QSYTQKLSEVFKVKVGAVSGCDKIFKNEKYGNLEFVTSITKRTNVLEKMVFVNKPNDYLLQHKDSLMQRK
HPF57_0920 211   QSYTQKLSEVFKVKVGAVSGCDKIFKNEKYGNLEFVTSITKRTNVLEKMVFVNKPNDYLLQHKDSLMQRK

                          290       300       310       320       330       340       350
HPP12_0908 281   IKKFNENNWFEWGRMHHISPKKRTYVNAKTRQKNPFFIHQCPNYDGSILALFPYNQNLDLQNLCDKLNAI
HPF16_0891 281   IKKFNENNWFEWGRMHHISPKKRIYVNTKTRQKNPFFIHQCPNYDGSILALFPYNQNLDLQNLCDKLNAI
HPF30_0429 281   IKKFNENNWFEWGRMHHISPKKRIYVNTKTRQKNPFFIHQCPNYDGSILALFPYNQNLDLQNLCDKLNAI
HPF32_0444 281   IKKFNEANWYKWGRMHYISPKKRIYVNTKTRQKNPFFIHQCPNYDGSILALFPYNQNLDLQNLCDKLNAI
HPF57_0920 281   IKKFNEANWYKWGRMHYISPKKRIYVNTKTHQKNPFFIHQCPNYDGSILALFPYNQNLDLQNLCDKLNAI

                          360       370       380
HPP12_0908 351   NWQELGFVCGGRFLFSQRSLENALLPKDFLNLG*
HPF16_0891 351   NWQELGFVCDGRFLFSQRSLENALLPKDFLNLG*
HPF30_0429 351   NWQELGFVCDGRFLFSQRSLENALLPKDFLNXG*
HPF32_0444 351   NWQELGFVCDGRFLFSQRSLENALLPKDFLNXG*
HPF57_0920 351   NWQELGFVCDGRFLFSQRSLENALLPKDFLNLG*
```

D (Type II, beta, 5'-TCGA)

```
                IV                    V      VI
         10        20        30        40        50        60        70
HPP12_0259   1  LHKVFIMEALECLKRIEKESIQTIYIDPPYNTKSSNFEYEDAHADYEKWIKEHLILAKAVLKQSGCIFIS
HPF16_0267   1  LHKVFIMEALECLKRIEKESIQTIYIDPPYNTKSSNFEYEDAHADYDKWIKEHLILAKAVLKQSGCIFIS
HPF30_1036   1  LHKVFIMEALECLKRIEKESIQTIYIDPPYNTKSSNFEYEDAHADYEKWIKEHLILAKAVLKQSGCIFIS
HPF32_0269   1  LHKVFIMEALECLKRIEKESIQTIYIDPPYNTKSSNFEYEDAHADYEKWIKEHLILAKAVLKQSGCIFIS

                   VII         VIII
         80        90       100       110       120       130       140
HPP12_0259  71  IDDNKMTEVKIIANEIFGTRNFLGTFITKQATRSNAKHINITHEYVLSYAKNKAFAPGFKILRTLLPIYA
HPF16_0267  71  IDDNKMAEVKIIANEIFGTCNFLGTFITKQATRSNAKHINITHEYVLSYAKNKAFAPGFKILRTLLPIYA
HPF30_1036  71  IDDNKMAEVKIIANKIFGMRNFLGTFITKQATRSNAKHINITHEYVLSYAKNKAFAPSFKILRTLLPVYA
HPF32_0269  71  IDDNKMAEVKIIANEIFGTRNFLGTFITKQATRSNAKHINITHEYVLSYAKNKAFAPSFKILRTLLPIYA

                          TRD
        150       160       170       180       190       200       210
HPP12_0259 141  KALKDLMRTIKNVFKQKGQTQAQLVLKEQIKELSQKEHFNFLKNYNLVDEKGEIYFAKDLSTLSNPRSVA
HPF16_0267 141  KPLKDLMRTIKNIFKQKGQAQAQLVLKEQIKELSQKEHFNFLKNYNLVDEKGEIYFAKDLSTPSNPRSVA
HPF30_1036 141  KPLKDLMRTIKNVFKQKGQAQAQLVLKEQIKELSQKEHFNFLKNYNLVDEKGEIYFAKDLSTPSNPRSVA
HPF32_0269 141  KPLKDLMRTIKNVFKQKGQAQAQLVLKEQIKELSQKEHFNFLKNYNLVDEKGEIYFAKDLSTPSNPRSVA

        220       230       240       250       260       270       280
HPP12_0259 211  IQEINLFLEPLKSRGWSSDEKLKELYYQNRLIFKNNRPYEKYYLKESQDNCLSVLDFYSRQGTKDLEKLG
HPF16_0267 211  IQEINLFLEPLKSRGWSSDEKLKELYYQNRLIFKNNRPYEKYYLKESQDNCLSVLDFYSRQGTKDLEKLG
HPF30_1036 211  IQEINLFLEPLKSRGWSSDEKLKELYYQNRLIFKNNRPYEKYYLKESQDNCLSVLDFYSRQGTKDLEKLG
HPF32_0269 211  IQEINLFLEPLKSRGWSSDEKLKDLYHQNRLIFKNNRPYEKYYLKESQDNCLSVLDFYSRQGTKDLEKLG

              X          I
        290       300       310       320       330       340       350
HPP12_0259 281  LKGLFKTPKPVALIKYLLLCSTPKDSIILDFFAGSGTTAQAVIEVNKDYYLNWSFYLCQKEEKIKNNPQA
HPF16_0267 281  LKGLFKTPKPVELIKYLLLCSTPKDSIILDFFAGSGTTAQAVIEVNKDYYLNWFFYLCQKEEKIKNNPQA
HPF30_1036 281  LKGLFKTPKPVALIKYLLLCSTPKDSIILDFFAGSGTTAQAVIEVNKDYYLNWSFYLCQKEEKIKNNPQA
HPF32_0269 281  LKGLFKTPKPVALIKYLLLCSTPKDSIILDFFAGSGTTAQAVIEVNKDYYLNWSFYLCQKEETIKNNPQA

                          III
        360       370       380
HPP12_0259 351  ASILKNGYKNTISDIMLLRLEKIIKSHEYEIL
HPF16_0267 351  ASILKNGYKNTISNIMLLRLEKIVKRSEYEIL
HPF30_1036 351  ASILKNGYQNTISNIMLLRLEKIIKSHEYEIL
HPF32_0269 351  ASILKNGYKNTISNIMLLRLEKIIKRSEYEIL
```

E (Type II, gamma,5'-TGCA)

```
                 10  X      20        30        40     I  50        60        70
HPP12_1523   1   MVSNTTLQKNLDAFYTHPKIARFCLDLLKNLINQNLGLDLNAFHFLEPSAGSGSFVDALKELGIADCIAL
HPF16_1447   1   MVSNTTLQKNLDAFYTHPKIARFCLDLLKDLINQNLGLDLNAFHFLEPSAGSGSFVGALKGLGIADCLAL
HPF30_1424   1   MVSNTTLQKNLDAFYTHPKIARFCLDLLKDLINQNLGLDLNAFHFLEPSAGSGSFVGALKGLGIADCLAL
HPF32_1440   1   MVSNTTLQKNLDAFYTHPKIARFCLDLLKDLINQNLGLGLNAFHFLEPSAGSGSFVGVLERLGIADCLAL
HPF57_1467   1   MVSNTTLQKNLDAFYTHPKIARFCLDLLKDLIHQNLGLGLNAFHFLEPSAGSGSFVGALKGLGIADCLAL

                 II      80 III    90       100IV     110  V     120   VI  130       140
HPP12_1523   71  DIAPKAQGIQKKDYLLELIEFNKKRVIIGNPPFGRRGKLALDFLNKSLNEVPIVAFILPNLFKRYSIQKR
HPF16_1447   71  DIAPKAQGIQKKDYLLELIGFNKKRIIIGNPPFGHRGKLALDFLNKSLNEAPIVAFILPNLFKRYSIQKH
HPF30_1424   71  DIAPKAQGIQKKDYLLELIEFNKKRVVIGNPPFGHRGKLALDFLNKSLNEAPIVAFILPNLFKRYSIQKH
HPF32_1440   71  DIAPKAQGIQKKDYLLELIEFNKKRIIIGNPPFGHRGKLALNFLNKSLNEAPIVAFILPNLFKRYSIQKH
HPF57_1467   71  DIAPKAQGIQKKDYLLELIEFNKKRIIIGNPPFGHRGKLALDFLNKSLNEAPIVAFILPNLFKRYSIQKH

                      VII  150       160       170VIII    180       190       200       210
HPP12_1523   141 IDKRAKLVLNADLEKNAFIFNERPYDVKCVFQIYMHKNIALNLKDERIIAPPKIRHNDFITYIHNNTPHT
HPF16_1447   141 IDKRAKLVLNADLEKNAFIFNERPYDVKCVFQIYMHKNIALNLKDERIIAPPKIRHNDFITYIHNNTPHT
HPF30_1424   141 IDKRAKLVLNADLEKNAFIFNERPYDVKCVFQIYMHKNIALNLKDERIIAPPKIRHNDFITYIHNNTPHT
HPF32_1440   141 IDKRAKLVLNADLEKNAFIFNERPYDVKCVFQIYMHKNIALNLKDERIIAPPKIRHNDFITYIHNNTPYT
HPF57_1467   141 IDKRAKLVLNADLEKNAFIFNERPYDVKCVFQIYMHKNIALNLKDERIIAPPKIRHNDFITYIHNNTPHT

                         220       230       240 TRD 250       260       270       280
HPP12_1523   211 LKYFNKEKYQWDFAVVRQGFYDYNEKITNANLLVKNRQYFFIKAHSKEALRIIHKIDFNKLAHKNTQVLG
HPF16_1447   211 LKYFNKEKYQWDFAVVRQGFYDYNEKITNANLLIKNRQYFFIKAHSKEALMIIHKIDFNKLAHKNTQVLG
HPF30_1424   211 LKYFNKEKYQWDFAVVRQGFYDYNEKITNANLLIKNRQYFFIKAHSKEALMIIHKIDFNKLAHKNTQVLG
HPF32_1440   211 LKYFNKEKYQWDFAVVRQGFYDYNEKITNANLLIKNRQYFFIKAHSKEALRIIHKIDFNKLAHKNTQVLG
HPF57_1467   211 LKYFNKEKYQWDFAVVRQGFYDYNEKITNANLLIKNRQYFFIKAHSKEALMIIHKIDFNKLAHKNTQVLG

                         290
HPP12_1523   281 FSTYN
HPF16_1447   281 FSTYDFVEEYCKLKEMHA*
HPF30_1424   281 FSTYDFVEEYCKLKEMHA*
HPF32_1440   281 FSTYDFVEEYCKLKEMHA*
HPF57_1467   281 FSTYDFVEEYCKLKEMHA*
```
